# Spatiotemporal Imaging of Catechol Aldehydes in Neural Tissue

**DOI:** 10.1021/jacsau.4c01249

**Published:** 2025-03-13

**Authors:** John M. Talbott, Rachel Wills, Rajendra Shirke, Leslie Hassanein, David Weinshenker, Monika Raj

**Affiliations:** † Department of Chemistry, Emory University, Atlanta, Georgia 30322, United States; ‡ Department of Human Genetics, Emory University School of Medicine, Atlanta, Georgia 30322, United States

**Keywords:** tissue imaging, catechol aldehydes, FLIM-FRET, spatiotemporal, neurodegenerative disorders

## Abstract

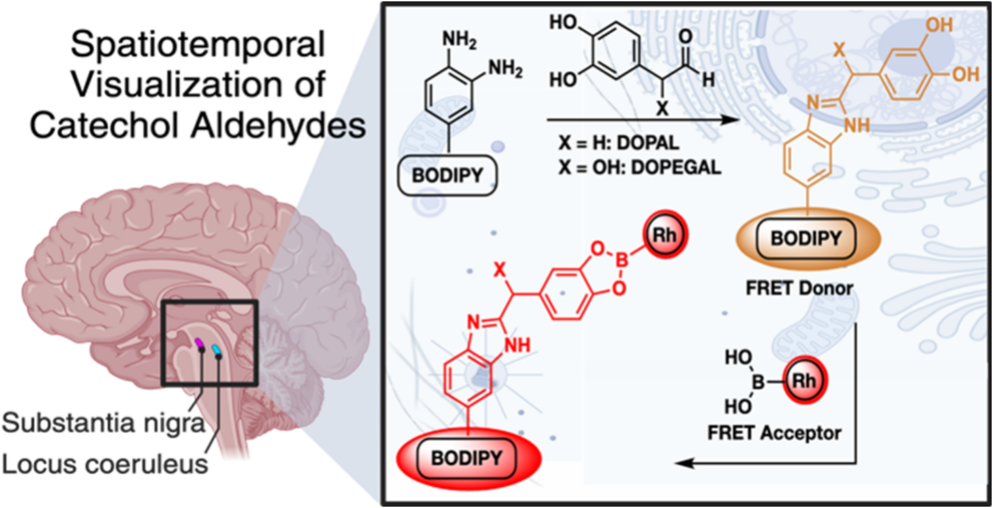

Catechol aldehydes (CAs), particularly 3,4-dihydroxyphenylacetaldehyde (DOPAL) and 3,4-dihydroxyphenylglycolaldehyde (DOPEGAL), are potently cytotoxic and have been implicated in pathogenesis of neurodegenerative disorders. Understanding the dynamics of CAs in the brain is crucial for elucidating neurodegenerative pathways. Herein, we present an innovative fluorescent sensor system designed for the selective imaging of CAs within cells and neural tissues. This system employs a dual-reaction trigger, utilizing o-phenylenediamine’s selectivity for aldehydes and phenylboronic acid for catechols, generating a specific Förster Resonance Energy Transfer (FRET) signal for CAs. Importantly, we have integrated fluorescence lifetime imaging microscopy (FLIM) with FRET (FLIM-FRET) to enhance detection accuracy while mitigating issues like spectral crosstalk and photobleaching. This dual-reaction FLIM-FRET system allows for the precise visualization of endogenous CAs in the substantia nigra and locus coeruleus of mice, the primary sites of CA production. Notably, this method represents a significant advancement in our ability to study these critical brain regions, as it uniquely enables the tracking of CAs spread across different parts of the brain, addressing a critical gap in the field, as no existing methods allow for such detailed localization of CAs across different brain regions. By enabling precise visualization of CAs within neural tissues, our method enhances understanding of their roles in disease progression.

Oxidative deamination of norepinephrine (NE) and dopamine (DA) by monoamine oxidase (MAO) in neurological tissue generates catechol aldehydes (CAs), specifically 3,4-dihydroxyphenylglycolaldehyde (DOPEGAL) and 3,4-dihydroxyphenylacetaldehyde (DOPAL), along with hydrogen peroxide.^1−3^ Under normal physiological conditions, these metabolites are detoxified by aldehyde dehydrogenase (ALDH) or aldehyde reductase (AR) to their corresponding carboxylic acid (DOPAC) or alcohol (DHPG).^4,5^ However, during oxidative stress, the activity of ALDH, AR, and other detoxifying enzymes becomes dysregulated, resulting in an accumulation of toxic metabolites DOPAL and DOPEGAL (Figure 1).^6,7^ These metabolites can react with biological nucleophiles, including proteins and nucleic acids, forming cross-linked structures such as neurofibrillary tangles (NFTs), causing irreversible structural and functional changes.^8−12^ The formation of these alterations involves (i) the creation of Schiff bases and thiazolidines with lysine and cysteine due to the aldehyde group,^13,14^ (ii) Michael-type additions of lysine and cysteine to the quinone obtained from the oxidation of catechol group,^15^ and (iii) Amadori rearrangement product with lysine or the N-terminus involving the α hydroxy of the aldehyde (Figure 1).^16^ These alterations contribute to various neurodegenerative diseases, including Parkinson’s disease (PD), Alzheimer’s disease (AD), Huntington’s disease, and schizophrenia.^17−21^ Currently, there is an ongoing investigation into the interplay between the roles of CAs and NFTs for the spread of neurodegenerative pathology in the brain.^22,23^ This distinction is crucial for therapeutic strategies and understanding disease progression. As of now, no method exists to visualize CAs in live cells and tissues, highlighting a significant gap in our ability to study their role in neurodegenerative diseases.

**Figure 1:**
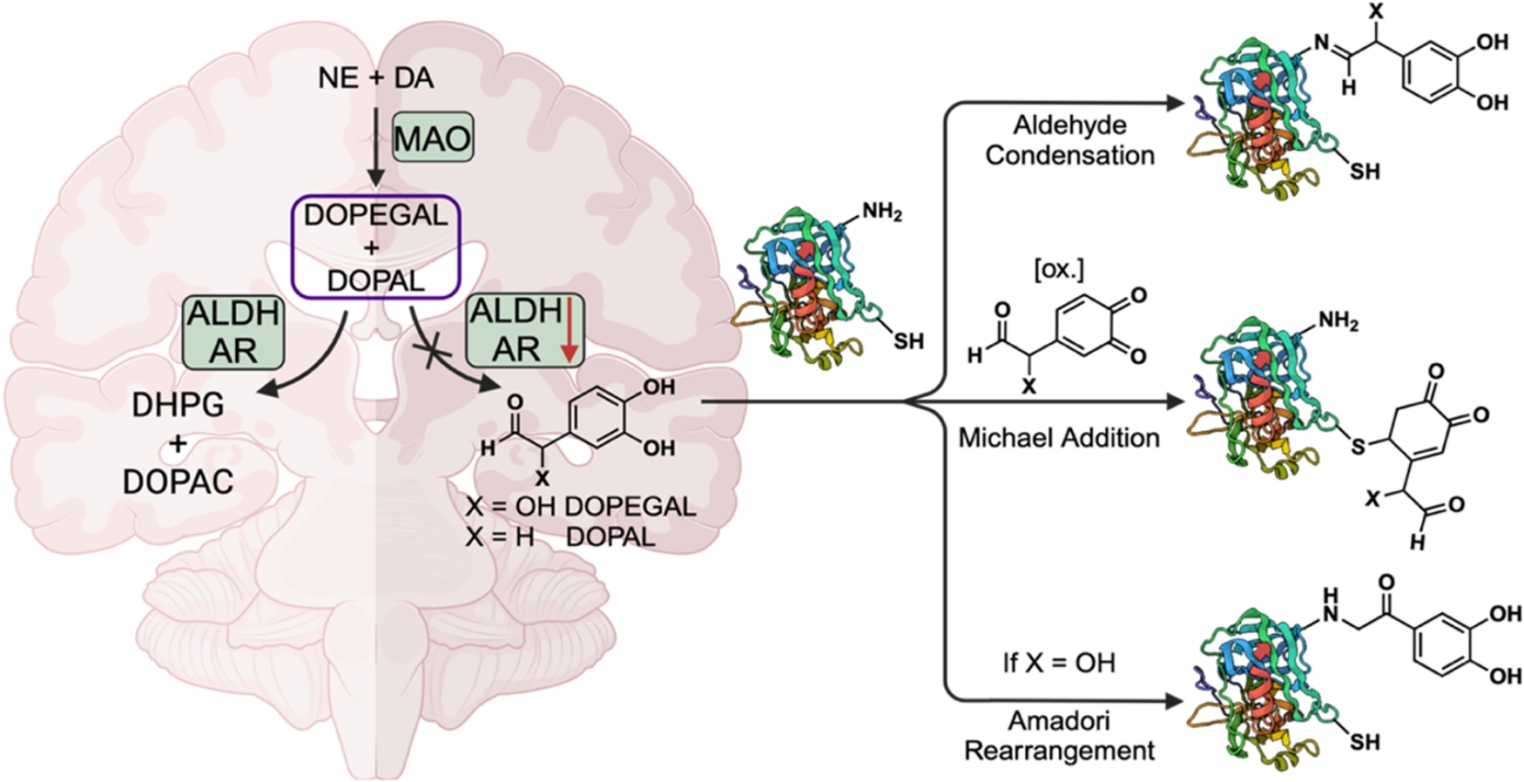
Metabolic pathway of norepinephrine (NE) and dopamine (DA) conversion to DOPEGAL and DOPAL by monoamine oxidase (MAO). These molecules are further processed by aldehyde dehydrogenase (ALDH) and aldehyde reductase (AR) to nontoxic dihydroxyphenylglycol (DHPG) and 3,4-dihydroxyphenylacetic acid (DOPAC). Dysregulation of ALDH and AR leads to accumulation of DOPEGAL and DOPAL, which can form covalent cross-links with biological nucleophiles leading to disease states.

Thus, we developed a novel fluorescent-based system for the specific visualization of catechol aldehydes to enhance spatiotemporal resolution compatible with tissue analysis. This dual-reaction Förster resonance energy transfer (FRET)-based sensor effectively addresses the challenges of detecting CAs within live cells and tissues. The sensor capitalizes on both the aldehyde and catechol components of CAs, allowing for their selective detection in the presence of other aldehydes and catechols. Our approach employs two key reactions, first, diamine-phenyl-BODIPY **1a** reacts with the aldehyde component to generate benzimidazole-BODIPY **2a**. Simultaneously, phenyl boronic acid-functionalized Rhodamine B **3a** interacts with the catechol component to produce RhoB-boronic ester **4a**. This dual-reaction mechanism results in a distinct FRET signal specific to catechol aldehydes (CAs), as benzimidazole-BODIPY complexes with noncatechol aldehydes (**2×**) cannot form a FRET pair, enabling the precise identification of CAs within live cells, even amidst other reactive functional groups (Figure 2).

**Figure 2:**
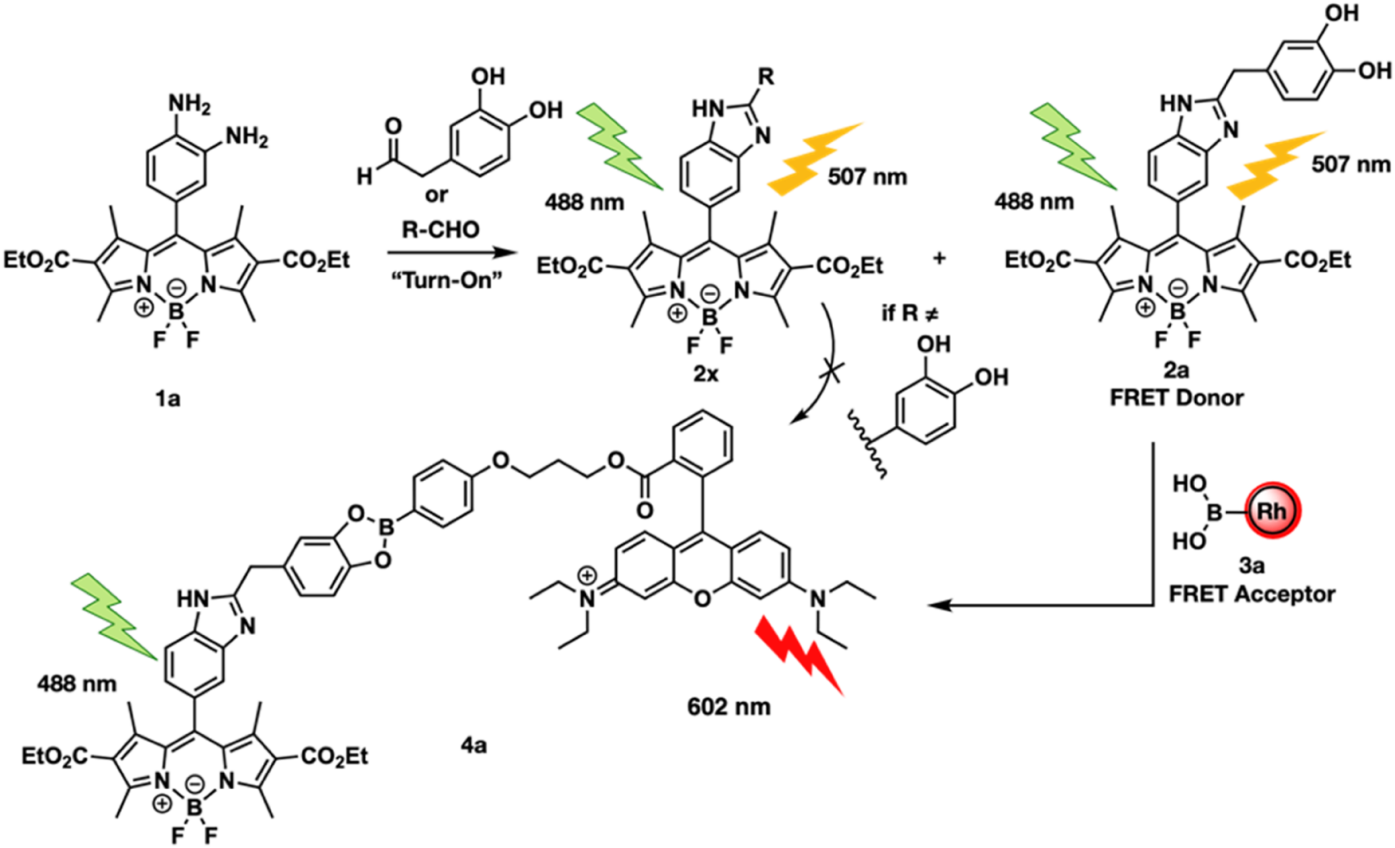
“Turn-On” fluorescence of probe **1a** with aldehydes and selective FRET formation of the catechol benzimidazole FRET donor **2a**. **2a** reacts with the boronic acid FRET acceptor **3a** to create the detectable FRET pair **4a**. Other aldehyde products (**2×**) cannot form a FRET pair.

To accurately visualize CAs in the brain, we employed Fluorescence Lifetime Imaging Microscopy (FLIM) combined with FRET (FLIM-FRET), which provides fluorescence lifetime measurements on a nanosecond scale.^24,25^ The fluorescence lifetime of the donor decreases when quenched by FRET interactions, allowing for sensitive detection of CAs. FLIM-FRET effectively mitigates various sources of interference, including spectral cross-talk, excitation intensity fluctuations, inner filtering, photobleaching, direct acceptor excitation, and detector sensitivity,^26^ making it highly suitable for live cell and tissue studies.^27,28^ Its lower detection limits enable the visualization of minute fractions of molecules engaged in FRET.^28^ Our study successfully detected endogenous catechol aldehydes (CAs) within live cells and monitored changes in relative levels of CAs in response to enzyme activators and inhibitors. Additionally, we demonstrated our dual-reaction FLIM-FRET system’s capability to identify CAs in the key brain regions, such as the substantia nigra (SN), where dopamine (DA) is converted to 3,4-dihydroxyphenylacetaldehyde (DOPAL), and the locus coeruleus (LC), where norepinephrine (NE) is converted to 3,4-dihydroxyphenylglycolaldehyde (DOPEGAL) by monoamine oxidase. To determine probe specificity, we examined CAs in the SN and LC of dopamine beta-hydroxylase knockout (DBH -/-) mice, which completely lack NE. We detected CAs in the SN of both DBH -/- and DBH +/- controls that have normal DA and NE levels. However, the CA signal in the LC of NE-competent DBH +/- mice was dramatically reduced in DBH -/- mice. These findings highlight the potential of our method for studying CA dynamics in the brain and their association with neurodegenerative diseases, providing valuable insights into their spread to other brain regions.

## Results and Discussion

### Development of FRET Probes for Catechol Aldehydes

In pursuit of our objectives, our laboratory has developed a probe utilizing 3,4-phenylenediamine linked to a boron-dipyrromethene (BODIPY) core, **1a**.^29,30^ Probe **1a** exhibits notable chemoselectivity for aldehydes in the presence of other biological metabolites. Upon reacting with DOPAL, probe **1a** produced DOPAL-benzimidazole-BODIPY **2a**, resulting in a distinctive fluorescence excitation at 488 nm. To selectively distinguish and identify catechol aldehydes from other reactive aldehydes, we devised a plan to leverage the distinctive catechol (1,2-dihydroxyphenyl) moiety of catechol aldehydes. Based on several literature reports indicating the swift and specific reactivity of boronic acids with catechols,^31,32^ we synthesized a phenylboronic acid-functionalized Rhodamine B **3a** (λ_ab_ = 566 nm) to serve as a FRET acceptor for the DOPAL-benzimidazole-BODIPY FRET donor **2a** (λ_em_ = 507 nm), by the formation of boronate ester **4a** with the catechol group of **2a**.

**Figure 3:**
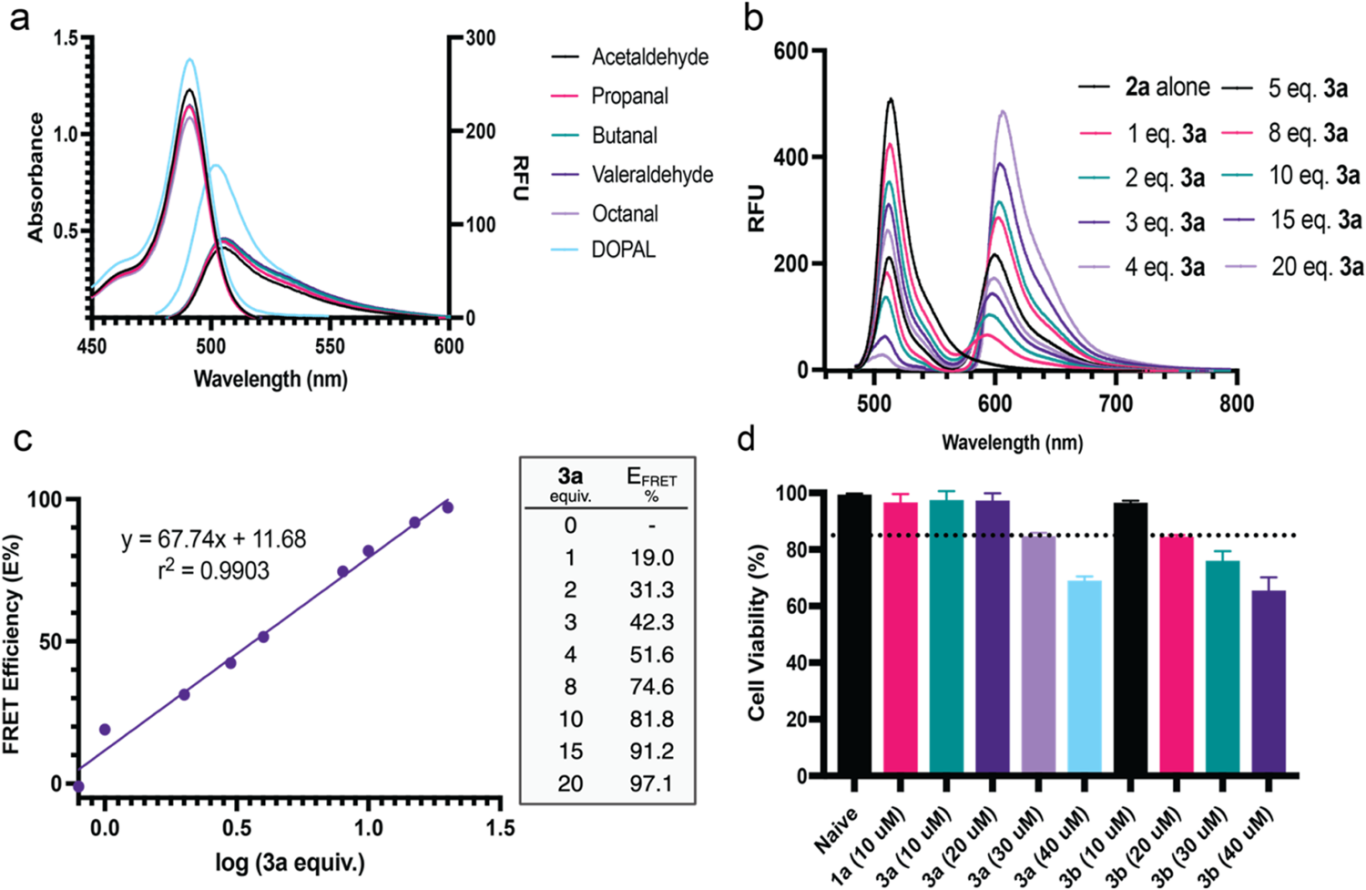
Fluorescent properties of probes **1a** and **3a**. (a) Excitation and emission of the benzimidazole products formed from the reaction of probe **1a** with varying aldehydes. RFU = relative fluorescent units. (b) Emission of FRET donor **2a** with varying equiv of FRET acceptor **3a**. (c) Calculated FRET efficiencies tabulated and plotted against equiv of **3a**. (d) Cell death of U-87 MG cells incubated with probes **1a**, **3a**, and **3b**. Dashed line represents 85% cell viability threshold. Errors bars represent standard deviation. All experiments were performed in triplicate.

**Scheme 1:**
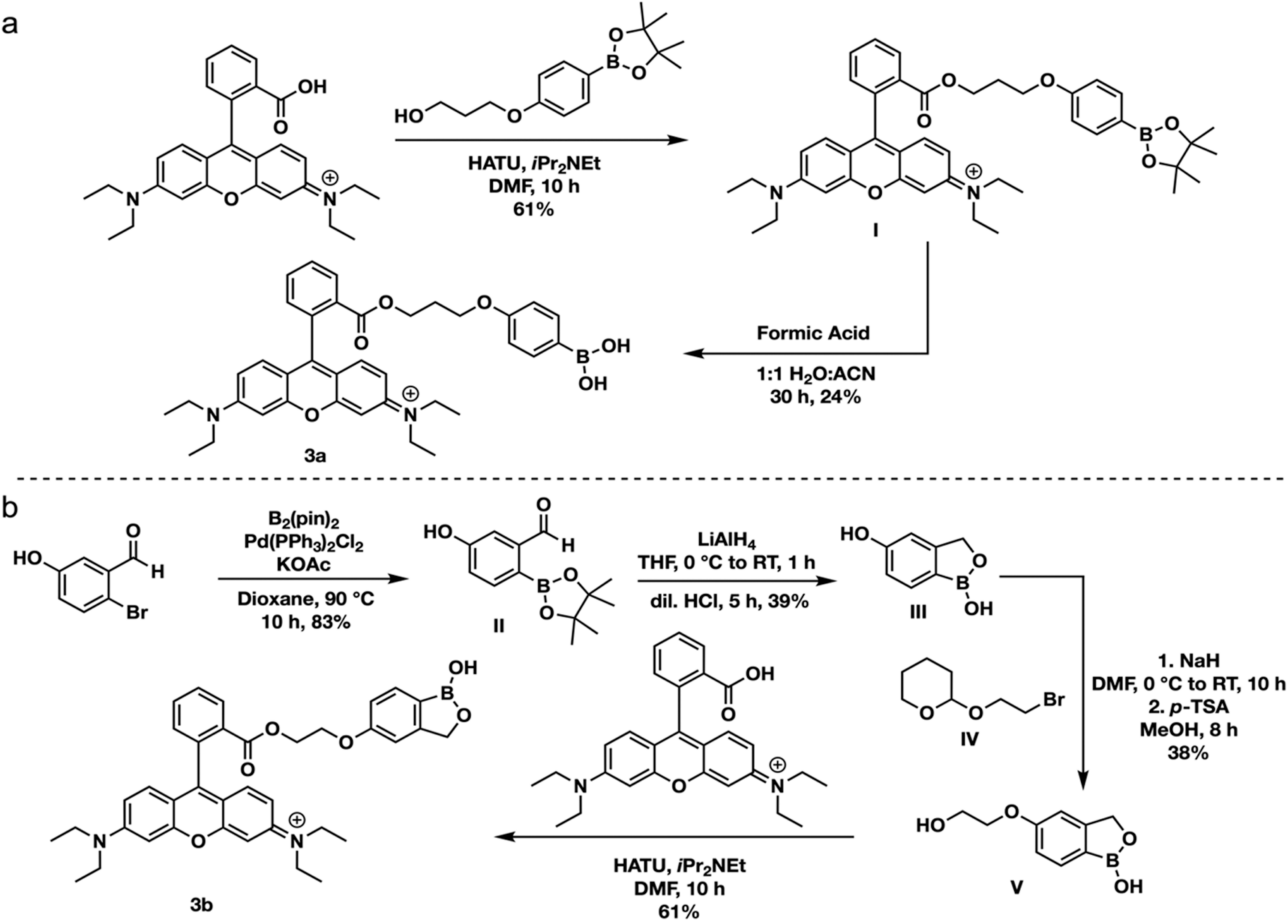
Synthesis of Rhodamine FRET Acceptors (a) Synthesis of **3a**. (b) Synthesis of **3b**.

Rhodamine B was selected as the FRET acceptor due to its established efficacy in FRET with BODIPY and compatibility in live cells and living systems.^33,34^ To further enhance the reaction rate with the catechol component of catechol aldehydes and to obtain the best response profile while enhancing the stability of the boronate ester under physiological conditions, we synthesized heterocyclic benzoxaboroles-functionalized Rhodamine B **3b** as a FRET acceptor.^35^ Synthesis of probe **3a** began with coupling Rhodamine B to a commercially available boronate ester before subsequent pinacol deprotection (Scheme 1a and Figure S1). Probe **3b** synthesis began with a Suzuki coupling of 2-bromo-5-hydroxybenzaldehyde with B_2_Pin_2_, which was converted to the benzoxaborole product using LiAlH_4_ before quenching in acidic conditions. Subsequent linker addition and coupling to Rhodamine B yielded **3b** (Scheme 1b and Figure S2). To assess the selectivity of these FRET pairs for catechol aldehydes, we initially incubated probe **1a** with various aldehydes, including acetaldehyde, propanal, butanal, valeraldehyde, octanal, and DOPAL, and recorded the absorbance and emission spectra of the corresponding benzimidazole products. Uniquely high absorbance and emission intensities were observed from DOPAL-benzimidazole-BODIPY **2a** compared to other aldehydes at the same concentrations (Figure 3a). This was further verified upon determination of the quantum yields of the BODIPY products. Benzimidazole **2a** exhibited a higher quantum yield of 0.29, compared to **2b**, which exhibited a quantum yield of 0.25 (Figure S4). Next, phenylboronic acid-functionalized Rhodamine B **3a** was added to DOPAL-benzimidazole-BODIPY **2a** at varying concentrations to determine its FRET efficiency. As expected, a dose-dependent increase in FRET efficiency was observed, with a maximum benchtop FRET efficiency of 97% at a 20 equiv of **3a** relative to **2a** (Figure 3b,c). No FRET signals were observed with other aldehydes and catechols tested, including dopamine and norepinephrine (Figure S5). This is due to the absence of both the aldehyde and catechol groups in the same structure. The selectivity toward catechol aldehydes stemmed from the chemoselective reaction between the 1,2-dihydroxyphenyl group of catechol aldehydes and the phenylboronic acid on the FRET acceptor, generating a boronate ester.^32^ Furthermore, the FRET signal showed no decrease in fluorescence over a 48-h incubation in media (Figure S5).

### Live Cell Compatibility and Selective Identification of Catechol Aldehydes by FLIM-FRET

To assess the operational efficacy of probes **1a, 3a**, and **3b** within live cells, we incubated them with U-87 MG, a human glioblastoma cell line commonly utilized in brain research,^36^ at various concentrations: **1a** (10 μM) and **3a** and **3b** (ranging from 10 to 40 μM) for 2 h, followed by flow cytometry analysis. The results revealed over 95% cell viability with 10 μM of **1a**, 20 μM of **3a** and 10 μM of **3b**. However, 30 μM of **3a** and 20 μM of **3b** led to more than 15% cell death (Figure 3d), Figure S6. Due to the high cell death observed with **3b**, we opted to proceed with 20 μM of **3a** for live cell studies. Furthermore, we wanted to verify that the probes were not localizing in subcellular compartments to ensure availability to form the FRET system in the presence of catechol aldehyde. Co-staining of **1a** and **3a** with MitoTracker and LysoTracker revealed that both probes were located in all non-nuclear compartments (Figure S7). To ensure selectivity in complex biological systems, we incubated cells with **1a** and **3a** alone or in the presence of glucose (20 mM) or catechol (1 mM). This would reveal if glucose (vicinal diol aldehyde when linear) or catechol (surrogate for catecholamine that cannot be oxidized to a CA) would not impact the fluorescent lifetime. No change in the lifetime population occurred, indicating the probe system’s selectivity for only catechol aldehydes (Figure S8). Additionally, we confirmed that increased concentration of reactive oxygen species (ROS) did not impact FLIM measurement (Figure S8).

**Figure 4:**
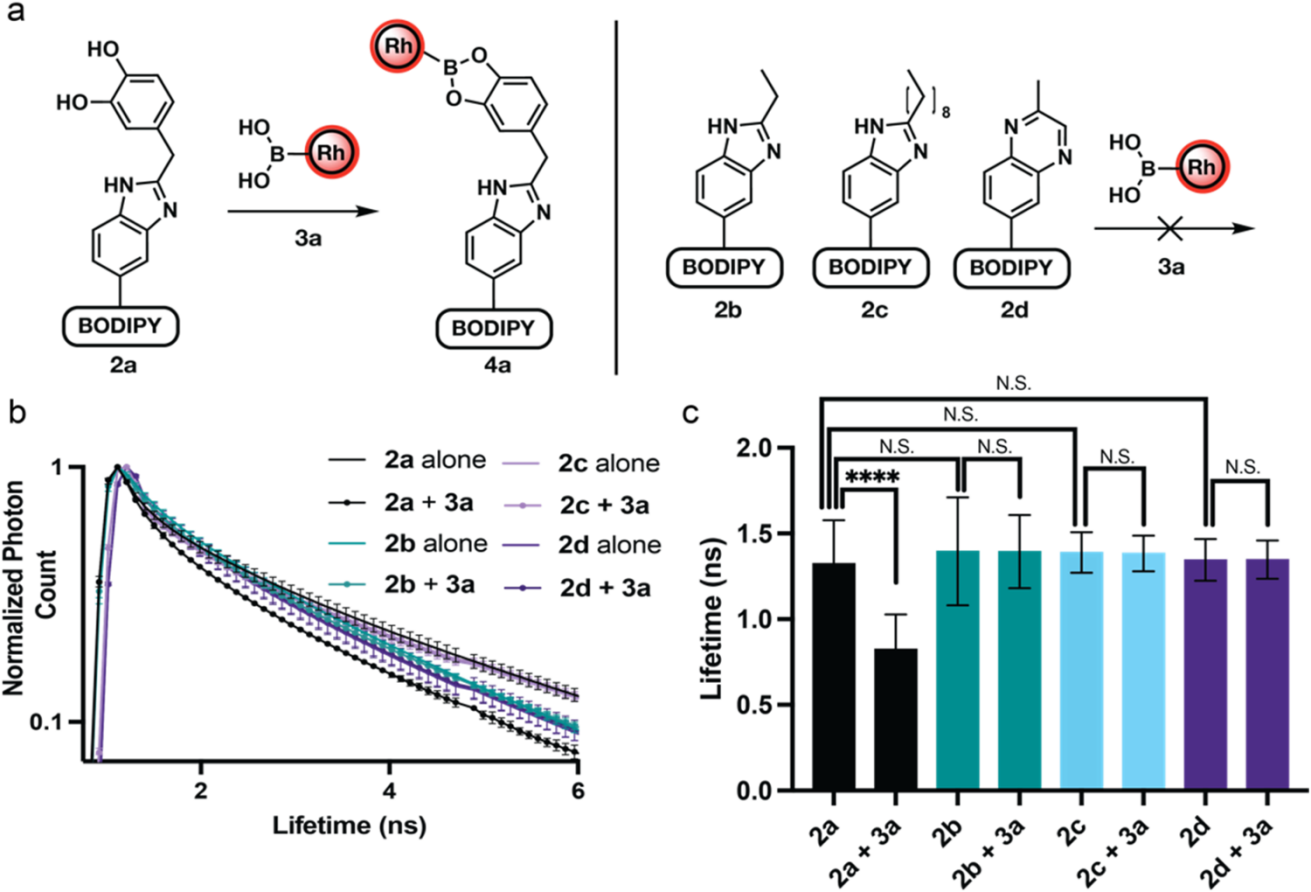
Selectivity of **2a** and **3a** FRET pair and resulting change in fluorescent lifetime. (a) Representation of FRET donor **2a** covalently interacting with FRET acceptor **3a** to give complex **4a** while **2b**-**2d** are unable to form an analogous product. (b) Lifetime decay curve of normalized photon counts of **2a**-**2d** alone and in the presence of **3a** in U-87 MG cells. Lifetimes were normalized to the largest photon count within each image. No change in lifetime decay curve for **2b**–**d** in the presence of **3a**. All experiments were performed in triplicate. (c) Average fluorescent lifetime of **2a**–**2d** alone and in the presence of **3a** in U-87 MG cells. Error bars represent standard deviation. Statistical significance determined by Student’s *t* test (*n* = 15), N.S. = not significant, **** = *p* < 0.0001.

Lastly, we sought to verify that only CAs would give a significant change in lifetime, while other aldehydes would give negligible change. Employing preformed DOPAL-benzimidazole-BODIPY **2a**, propanal-benzimidazole-BODIPY **2b**, decanal-benzimidazole-BODIPY **2c**, and methylglyoxal (MGO)-quinoxaline-BODIPY **2d**, we examined the capability of phenylboronic acid-functionalized Rhodamine B **3a** to exhibit FLIM-FRET signal within live cells. U-87 MG cells treated with **2a** (10 μM) for 2 h, were exposed to **3a** (20 μM) for 20 min, and the FLIM-FRET signal was measured using a Leica Stellaris 8 microscope (Figure 4). Inside U-87 MG cells, the DOPAL-benzimidazole-BODIPY **2a** had an average lifetime of 1.321 ns while the propanal-benzimidazole-BODIPY **2b** had an average lifetime of 1.436 ns. When probe **3a** was introduced to cells, **2a** showed a reduction in average fluorescent lifetime to 0.824 ns, while **2b** had no noticeable change in the fluorescence lifetime with an average of 1.394 ns (Figures 4 and S9). Decanal-benzimidazole-BODIPY **2c** and methylglyoxal (MGO)-quinoxaline-BODIPY **2d** also displayed the same fluorescence lifetime as the other aldehyde products **2a** and **2b** and showed no change in lifetime with the addition of **3a** (Figures 4 and S9). Decrease in fluorescence lifetime was observed exclusively with catechol aldehydes (DOPAL), with a FRET efficiency (E%) of 38.1%, thus highlighting its potential in selectively detecting catechol aldehydes within live cellular environments.

### Live Cell Imaging and Monitoring of Exogenous Catechol Aldehyde Levels in Preclinically Relevant Disease Models

**Figure 5:**
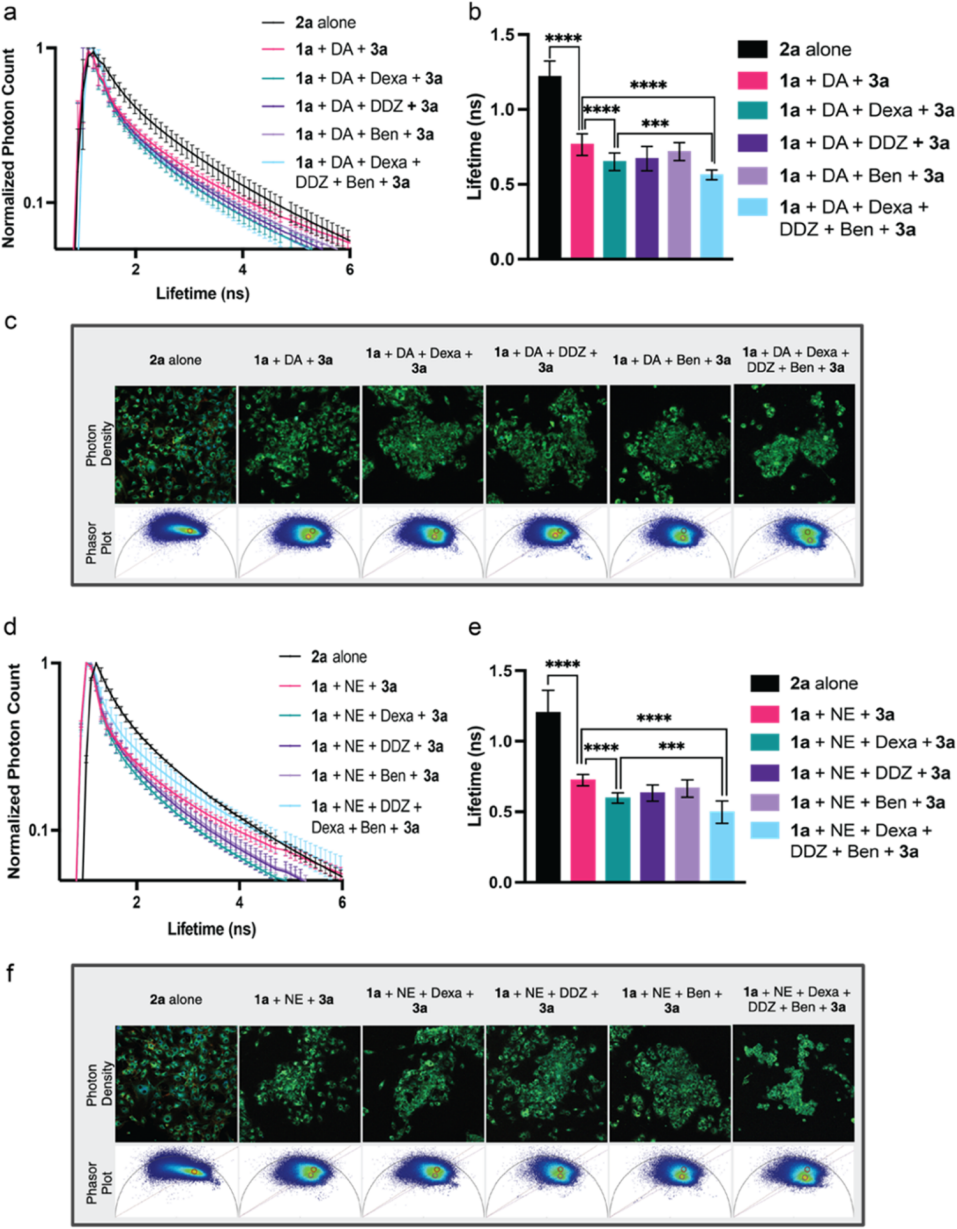
Live cell FLIM-FRET analysis of U-87 MG cells dosed with exogenous dopamine (DA) and norepinephrine (NE). (a) Lifetime decay curve of the normalized photon counts for **1a** reacting with DOPAL in the presence of **3a** in U-87 MG cells treated with exogenous DA, Dexa, DDZ, and Ben individually and in combination. Lifetimes were normalized to the largest photon count within each image. All experiments were performed in triplicate. (b) Average fluorescent lifetime of **1a** reacting with DOPAL in the presence of **3a** in U-87 MG cells in the presence of exogenous DA, Dexa, DDZ, and Ben individually and in combination. All experiments were performed in triplicate. (c) Representative image of photon density of U-87 MG cells and corresponding phasor plots. (d) Lifetime decay curve of the normalized photon count for a donor complex between for **1a** and DOPEGAL in the presence of **3a** in U-87 MG cells treated with exogenous NE, Dexa, DDZ, and Ben individually and in combination. All the experiments were performed in triplicate. (e) Average fluorescent lifetime of a donor complex between **1a** and DOPEGAL in the presence of **3a** in U-87 MG cells in the presence of exogenous NE, Dexa, DDZ, and Ben individually and in combination. All experiments were performed in triplicate. (f) Representative image of photon density of U-87 MG cells and corresponding phasor plots. Error bars represent standard deviation. Statistical significance determined by Student’s *t* test (*n* = 15), *** = *p* < 0.001, **** = *p* < 0.0001.

To evaluate the efficacy of our dual-reaction FLIM-FRET system in detecting natural DOPAL production during metabolic processes within live cells, we treated U-87 MG cells with dopamine (DA) (for DOPAL production), dexamethasone (Dexa) (an MAO activator),^37^ diadzin (DDZ),^38^ and benomyl (Ben)^39^ (ALDH2 and ALDH inhibitors, respectively), for 15 min. Subsequently, the cells were cotreated with 10 μM of probe **1a** for 2 h, before treatment with 20 μM of FRET acceptor **3a** for 15 min (Figure 5a). The addition of dopamine resulted in a significant decrease in the fluorescence lifetime of the FRET donor **2a** (0.765 ns, *E*% = 37.2%) (Figures 5a,b and S10) due to the increased concentration of DOPAL generated in the cells. The decrease in lifetime directly correlates to the reactivity of complex **2a** with probe **3a** to form the FLIM-FRET system. Addition of MAO activator, Dexa resulted in further lowering of fluorescence lifetime (0.650 ns, *E*% = 46.6%) due to further stimulation of DOPAL through the activation of MAO (Figures 5a,b and S10). The addition of DDZ (0.672 ns, *E*% = 44.8%), and Ben (0.712 ns, *E*% = 41.1%) further lowered the fluorescence lifetime of **2a** by blocking the clearance pathway of DOPAL through the inhibition of ALDH enzymes. The change in lifetimes can be visualized by phasor plots with the formation of a new cluster of lifetimes (Figures 5c and S10). This decrease is primarily attributed to the increased DOPAL production from dopamine and its accumulation due to the inhibition of DOPAL conversion to 3,4-dihydroxyphenylacetic acid (DOPAC),^4,5^ revealing that the combined treatment of DA/Dexa/DDZ/Ben yielded a significant decrease in the fluorescence lifetime of the **2a** to 0.564 ns (Figures 5a,b and S10). The results showed an increase in the formation of **4a** based on the increased presence of DOPAL in the cellular system from the activity of MAO activator and ALDH inhibitors, allowing for selective detection of CAs.

To investigate whether the acceleration of endogenous norepinephrine (NE) metabolism and DOPEGAL production^5^ would yield similar effects, we incubated live U-87 MG cells with NE, followed by MAO activator and/or ALDH inhibitors along with the donor probe **1a** followed by acceptor probe **3a** (Figures 5d–f and S11). Similar to observations with dopamine metabolism, a decrease in the fluorescence lifetime of the donor **1a** with NE (0.723 ns, *E*% = 39.8%) was observed in the presence of Dexa (0.597 ns, *E*% = 50.3%), DDZ (0.632 ns, *E*% = 47.4%), or Ben (0.666 ns, *E*% = 44.6%), while a much larger decrease in the donor lifetime (0.541 ns, *E*% = 54.1%) was observed in cells treated with a combination of all drugs (Dexa/DDZ/Ben) (Figure 5d–f). These results indicate the formation of FRET system through the reaction of DOPEGAL with probe **1a**, followed by the subsequent reaction with FRET acceptor **3a** inside live cells (Figures 5d–f and S11). These findings underscore the sensitivity of our dual-reaction trigger FLIM-FRET system in detecting alterations in catechol aldehyde levels, indicating its potential utility across diverse pathogenic conditions.

### Live Cell Imaging and Monitoring of Endogenous DOPAL and DOPEGAL Levels

**Figure 6:**
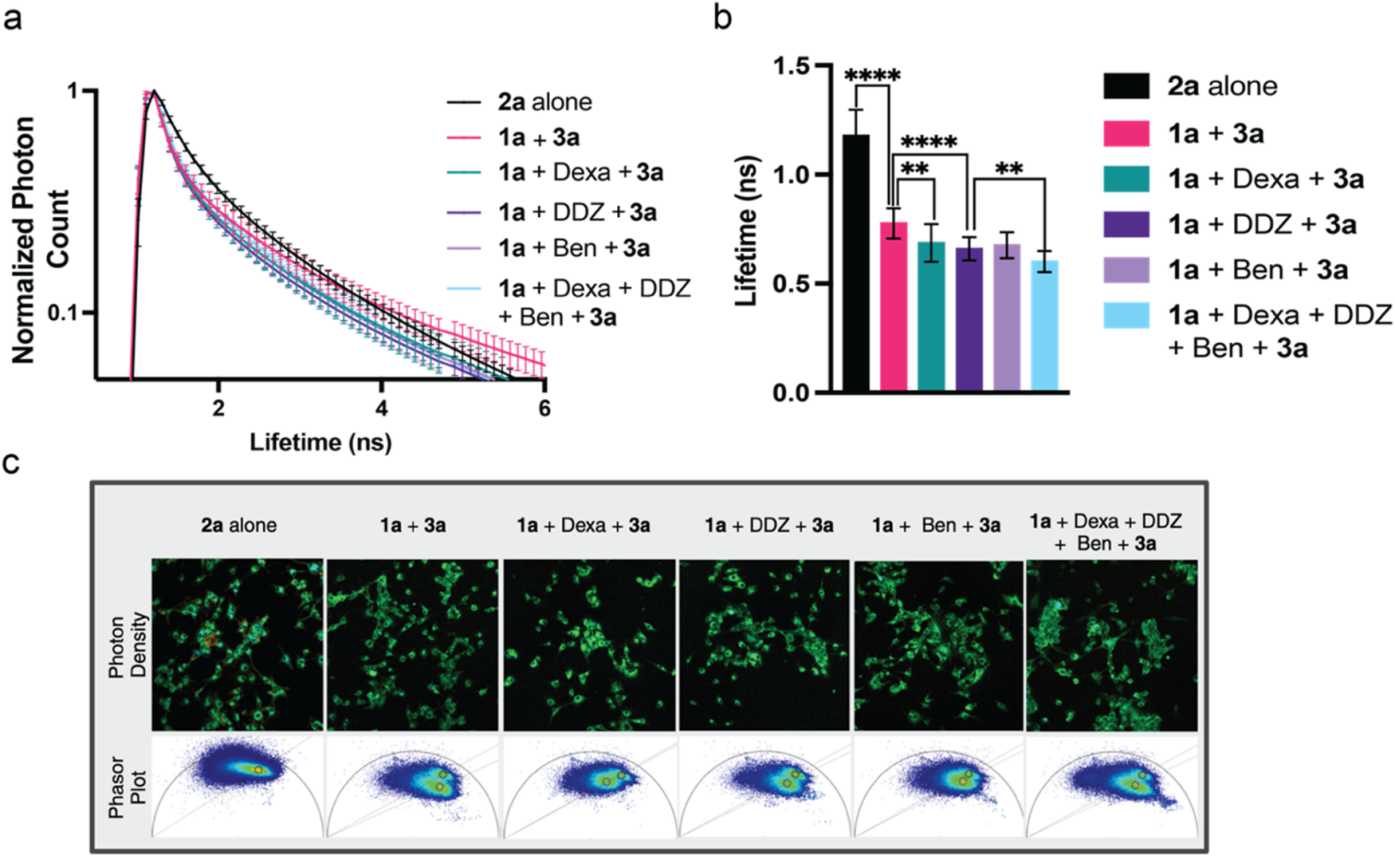
Live cell FLIM-FRET analysis of U-87 MG cells for endogenous DOPAL and DOPEGAL detection. (a) Lifetime decay curve of the normalized photon counts for complexes between **1a** and DOPAL and DOPEGAL in the presence of **3a** in U-87 MG cells treated with MAO activator (Dexa) and ALDH inhibitors (DDZ and Ben) individually and in combination. Lifetimes were normalized to the largest photon count within each image. All experiments were performed in triplicate. (b) Average fluorescent lifetime of complexes between **1a** and DOPAL and DOPEGAL in the presence of **3a** in U-87 MG cells treated with MAO activator (Dexa) and ALDH inhibitors (DDZ and Ben) individually and in combination. All experiments were performed in triplicate. (c) Representative image of photon density of U-87 MG cells and corresponding phasor plots. Error bars represent standard deviation. Statistical significance determined by Student’s *t* test (*n* = 15), ** = *p* < 0.01, **** = *p* < 0.0001.

Building upon these promising results, we proceeded to detect changes in endogenous levels of catechol aldehydes, including both DOPAL and DOPEGAL, within live cells. U-87 MG cells were treated with Dexa, DDZ, and Ben, either individually or in combination, followed by treatment with 10 μM of probe **1a** for 2 h, then 20 μM of FRET acceptor **3a** for 15 min. A decrease in the fluorescence lifetime of the donor **2a** (0.782 ns, *E*% = 33.5%) was observed in the presence of Dexa (0.691 ns, *E*% = 41.2%), DDZ (0.663 ns, *E*% = 43.6%), or Ben (0.681 ns, *E*% = 42.1%), with a much larger decrease in the donor lifetime (0.601 ns, *E*% = 48.2%) was observed in cells treated with all drugs (Dexa/DDz/Ben) in combination (Figures 6 and S12). These results affirm the high sensitivity of our FRET probes **1a** and **3a**, qualifying them for the identification of natural aldehyde production in both diseased and nondiseased cellular states.

### Detection and Visualization of CAs in Neural Tissue

**Figure 7:**
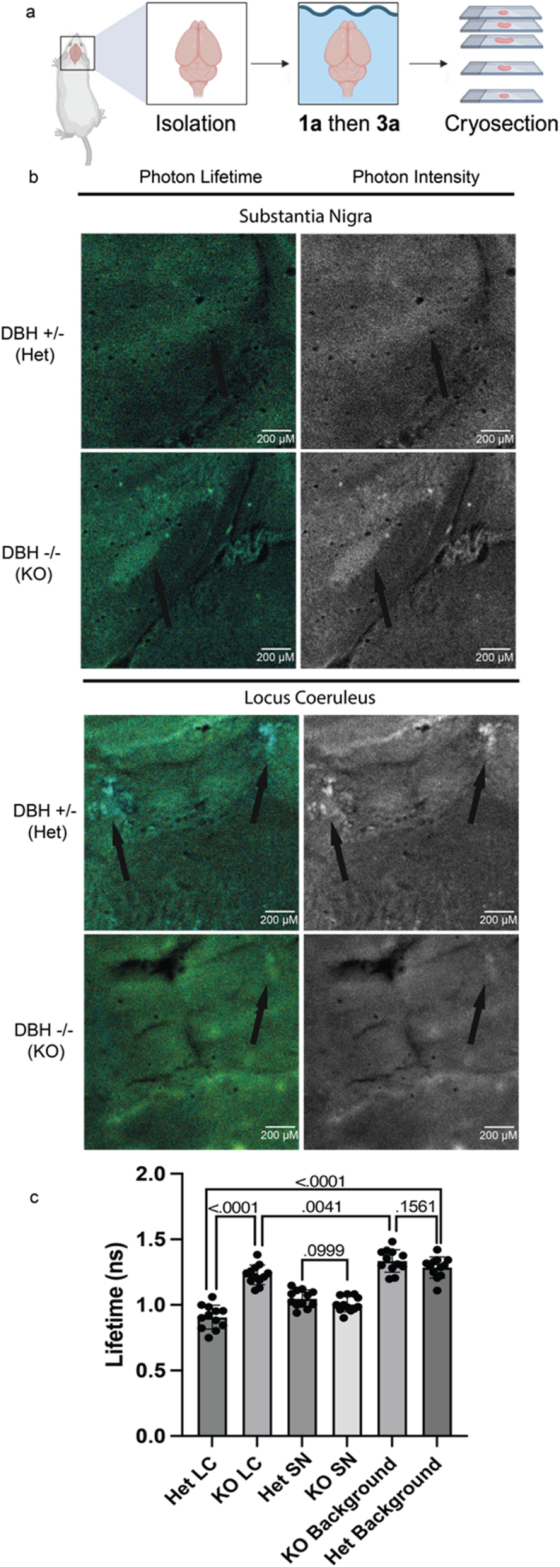
Tissue imaging of dopamine β-hydroxylase (DBH) heterozygous and knockout mice with probe **1a** and **3a**. (a) Schematic of tissue imaging protocol in DBH heterozygous (DBH+/-) and knockout (DBH −/−) mice. (b) FLIM photon lifetime and photon intensity of DBH +/- (Het) and DBH −/– (KO) mice brain SN and LC tissue. Photon lifetime is represented as a heat map, where light blue pixels represent lower fluorescent lifetime which are alternatively visualized as white hot spots in pixel intensity images. The location of the probe signals is consistent with the neuroanatomical location of the SN as well as the LC as a bilateral structure just below the lateral edges of the 4th ventricle in the pons. (c) Lifetimes (ns) from segmented subregions of each image. Background is defined as non SN and LC region. Error bars represent standard deviation. Statistical significance determined by Student’s *t* test (*n* = 12), *p* values shown on graph.

Encouraged by these outcomes, we applied our dual-reaction FRET-based sensor to investigate the roles of CAs in neurodegenerative pathology across various brain regions. Our focus was on visualizing catechol aldehydes (CAs) specifically in the substantia nigra (SN), which produces DA and DOPAL, and the locus coeruleus (LC), which produces NE and DOPEGAL.^22,40,41^ To evaluate the effectiveness and specificity of our probes in detecting CAs, we compared CA signals in the SN and LC of dopamine beta-hydroxylase knockout (DBH −/−) mice, which lack the enzyme necessary to convert DA to NE.^42−44^ This absence of DBH means that these knockout mice cannot produce DOPEGAL in the LC, creating a clear framework for assessing the specificity of our detection system. After sacrifice, whole brains were incubated with probe **1a** for 16 h, followed by probe **3a** for 8 h, then cryosectioned to isolate slices containing the SN and LC and subsequent imaging (Figures 7a and S13). As expected, DOPAL was observed in the SN region for both the DBH (+/-) and DBH knockout (DBH −/−) mice. However, the most significant insights were derived from analyzing the photon intensity from the fluorescent lifetime heat map, which provided crucial spatiotemporal information regarding DOPAL (Figures 7b and S13).

Notably, CA was mainly visible in the LC of DBH +/- mice, which have normal NE content, due to the formation of DOPEGAL. Furthermore, region of interest (ROI) analysis revealed a significantly shorter fluorescence lifetime (0.906 ns average from two ROIs per image of tissue slice, three tissue slices per mouse, from two mice; *n* = 12) compared to DBH -\- mice due to the inability to synthesize NE (1.230 ns average from two ROIs per image of tissue slice, three tissue slices per mouse, from two mice; *n* = 12, *p* < 0.0001). The photon intensity further elucidated the spatiotemporal distribution of CAs (Figures 7b and S13). Although the DBH knockout LC displayed a slight decrease in lifetime compared to neighboring subregions not associated with CA (1.335 ns, *p* = 0.004), this can likely be attributed to direct conversion of DA to DOPAL in the LC (Figure 7c). Additionally, the probe signal locations were consistent with the neuroanatomical positioning of the LC, identified as a bilateral structure just below the lateral edges of the fourth ventricle in the pons.^45,46^ Furthermore, the DBH +/- and KO SN average lifetimes were not statistically different (Figure 7c), further proving the system’s ability to detect CAs in native murine brain tissue. Given that DOPEGAL levels are elevated in the LC of Alzheimer’s disease brains and contribute to the aggregation of Tau protein and its propagation to the forebrain^20,22,47^ in animal models, our probes have the potential to enhance our understanding of the dynamics of catechol aldehydes and their role in spreading pathology to other areas of the brain. This insight could be crucial for elucidating the mechanisms underlying the onset and progression of Alzheimer’s disease.

## Conclusions

In this research endeavor, we have successfully developed a FRET donor–acceptor pair coupled with FLIM, enabling the selective detection of catechol aldehydes within living systems. Our dual-reaction FLIM-FRET system requires the presence of both an aldehyde group and a catechol group on the same molecule for efficient FRET signaling, demonstrating high selectivity for catechol aldehydes even in the presence of other reactive functional groups. The reaction between 3,4-diamino-BODIPY and catechol aldehyde, DOPAL, yields DOPAL-benzimidazole-BODIPY (**2a**), resulting in a significant increase in fluorescence. Meanwhile, the interaction of the catechol group of DOPAL-benzimidazole-BODIPY with phenylboronic acid-functionalized Rhodamine B (**3a**) produces a boronate, facilitating effective FRET signaling between the donor and acceptor. By employing FLIM, we effectively mitigate interference from fluctuations in excitation intensity, inner filtering, photobleaching, spectral cross-talk, and direct acceptor excitation. Our system has demonstrated the ability to detect both exogenous and endogenous catechol aldehydes, including DOPAL and DOPEGAL, in live cells. We further utilized our probes to investigate the roles of CAs in the development of neurodegenerative pathology by visualizing CAs in the SN and the LC of both control (DBH+/-) and DBH knockout (DBH −/−) mice, highlighting their utility in biomedical research.

Collectively, our probes provide a powerful tool for investigating the dynamics of catechol aldehydes in biological systems, free from interference of other biological metabolites. This capability paves the way for a deeper understanding of CA dynamics and their role in the progression of neurodegenerative diseases, including their potential to spread and influence pathological processes in other brain regions. Our findings lay a crucial foundation for future investigations into catechol aldehyde-related pathogenesis in neurological, cardiovascular, and metabolic disorders.

## Methods

### Synthesis

#### Procedure for Rhodamine Boronate Ester (**I**)

An oven-dried 25 mL RBF was charged with Rhodamine B (1.0 equiv), HATU (2.0 equiv), *i*Pr_2_NEt (2.0 equiv) in dry dimethylformamide (DMF) at RT. After 15 min, boronate ester (1.3 equiv) was added and stirred at RT for 10 h. The reaction progress was monitored by thin-layer chromatography (TLC). Upon completion, the reaction mixture was dissolved in DCM. The organic layer was washed with water and separated. The aqueous layer was back extracted with DCM. The combined organic layers were washed with saturated aqueous NaCl, dried over MgSO_4_, and concentrated under reduced pressure. The crude residue was purified by silica gel column chromatography using DCM/MeOH (9:1) as eluent to afford coupled product. This compound was isolated as pink solid.

#### Procedure for Rhodamine Boronic Acid (**3a**)

The Rhodium B boronate ester **I** (1.0 equiv) was dissolved in ACN:H_2_O (1:1) followed by the addition of formic acid (10 equiv) at RT and stirred for 30 h. The reaction progress was monitored by TLC. Upon completion, the reaction mixture was dissolved in DCM. The organic layer was washed with water and separated. The aqueous layer was back extracted with DCM. The combined organic layers were washed with saturated aqueous NaCl, dried over MgSO_4_, and concentrated under reduced pressure. The crude residue was purified by silica gel column chromatography using DCM/MeOH (9:1) as eluent to afford **3a**. This compound was isolated as pink solid.

### Cell Culture

Human U-87 MG cells were cultured in Dulbecco’s modified Eagle’s medium (DMEM) containing 10% fetal bovine serum (FBS), 1% (V/V) penicillin/streptomycin. Cells were maintained in an incubator at 37 °C with a 5% CO_2_/air environment.

### Cellular Studies

#### Flow Cytometry

Cells were grown in 60 mm × 15 mm Nunclon dishes. Stock solutions of compounds were prepared in dimethyl sulfoxide (DMSO) before being diluted to the final desired concentration in 4 mL of culture media. Cells were placed in the incubator for treatment for 2 h. Cells were then detached with trypsin and stained using Annexin V/PI following the manufacturer’s protocol (BioLegend cat: 640928). Briefly, the cells were detached with trypsin, washed twice with PBS, resuspended in 100 μL of cold AV binding buffer. Then, cells were stained with 10 μL of Pacific Blue Annexin V for 10 min, followed by addition of 10 μL of propidium iodide solution for 10 min. After adding 400 μL of Annexin V binding buffer to each tube, cells were analyzed via flow cytometry within 1 h to quantify cell death utilizing a BD FACSymphony A3 Cell Analyzer. FlowJo software was used to analyze cytometry data.

#### Colocalization Studies

Cells were plated in an IBIDI 8-well glass bottom chamber at a density of 25,000 cells per well in media and allowed to adhere overnight at 37 °C, 5% CO_2_. Fresh 10 mM stock solutions of probes **1a** and **3a** were prepared in DMSO on the day of experimentation. Working solutions of all compounds in media were prepared the day of experimentation. Culture media was removed from wells and 200 μL of 10 μM **1a** was added to desired wells and placed in an incubator for 1.5 h. Media was removed from wells and 200 μL of 20 μM **3a** was added to desired wells and placed in an incubator for 15 min. Then, probe media was removed, and cells were washed with 200 μL of PBS for 5 min in the incubator (repeated 3×). Cells were then stained with either LysoTracker RED DND-99 (Thermo Fisher, L7528) or Mitotracker FM (Thermo Fisher, M22425) was added according to manufacturer protocol, and cells were incubated for 20 min before staining media was removed and cells were washed with 200 μL of PBS for 5 min (repeated 3 times). PBS was removed and replaced with 200 μL of fresh media followed by immediate imaging. Five images were captured for each well. Colocalization analysis for Pearsons’ R (*R*) and Mander’s Colocalization Coefficient (MCC) were conducted using the EzColocalization Plugin for ImageJ.^48^

#### Live Cell Imaging

U-87 MG cells were plated in an IBIDI 8-well glass bottom chamber at a density of 25,000 cells per well in media and allowed to adhere overnight at 37 °C, 5% CO_2_. Fresh 10 mM stock solutions of probes **1a**, **2a**, and **3a** were prepared in DMSO on the day of experimentation. All other compounds were prepared fresh weekly in DMSO. Working solutions of all compounds in culture media were prepared the day of experimentation to final concentrations of 1 mM DA, 1 mM NE, 50 μM DDZ, 25 μM Dexa, and 50 μM Ben. Media was removed from wells, and 200 μL of the drugs was added to desired wells. Cells were placed in an incubator for 15 min, followed by removal of the media. Solutions of probe **1a** in combination with or without drugs or **2a** alone were added to desired cells and placed in an incubator for 2 h. Next, dosage media was removed, and cells were washed with 200 μL of PBS for 5 min in the incubator. PBS was removed, and 200 μL of the 20 μM solution of either probe **3a** or media was added and placed in the incubator for 15 min, followed by removal of the dosage media and washing with 200 μL of PBS for 5 min in the incubator (repeated 3 times). PBS was removed and replaced with 200 μL of standard culture media followed by immediate imaging. Five images were captured for each well utilizing a Stellaris 8 Leica DMi8 microscope (20× objective) with fast lifetime contrast (FALCON) module. Samples were excited using an 80 MHz pulsed white light laser tuned to 488 nm for both intensity and fluorescence lifetime measurements. Emitted photons were detected using HyD X (GaAsP hybrid photocathode). Image resolution of 1024 × 1024 was utilized and acquisition was recorded for 50 frames. This process was repeated in triplicate on separate days with different cell passage numbers for each experiment. Fluorescent lifetimes were determined by LASX Software. Statistical analysis was conducted via Student’s *t* test (*n* = 15). An outlier test was performed to remove any extraneous data points. Error bars represent standard deviation.

### Animal Studies

Adult male and female dopamine beta-hydroxylase knockout (Dbh -/-) mice, maintained on a mixed 129/SvEv and C57BL/6 J background as previously described,^42,43^ were used in this study. Heterozygous (Dbh +/−) littermates were used as controls because their behavior and catecholamine levels are indistinguishable from wild-type (Dbh +/+) mice.^44,49^ Animals were maintained on a 12:12 light:dark cycle (lights on at 0700), and food and water were *ad Libitum*. Mice were anesthetized with isoflurane and euthanized by rapid decapitation. Mouse brains were rapidly dissected on ice and flash-frozen in isopentane on dry ice. Samples were stored at −20 °C for 24 h. Brains were transferred to 5 mL centrifuge tubes containing 10 μM probe **1a** in PBS and allowed to diffuse for 16 h in a 4 °C refrigerator. Solution was removed and replaced with 20 μM probe **3a** in PBS and allowed to diffuse for 8 h in a 4 °C refrigerator. Solution was removed and brains were fixed with paraformaldehyde for 8 h. Brains were embedded in OT medium (Tissue-Tek) and sectioned by cryostat into 60 μM thick coronal sections at the level of the LC or SN. Sections were immediately transferred to glass Superfrost Plus slides which were then coverslipped with Fluoromount-G (Southern Biotech, Birmingham, AL) and allowed to dry before imaging. Images were captured for each slide utilizing a Stellaris 8 Leica DMi8 microscope (5× objective). Samples were excited using an 80 MHz pulsed white light laser tuned to 488 nm for both intensity and fluorescence lifetime measurements. Emitted photons were detected using HyD X (GaAsP hybrid photocathode). Image resolution of 2048 × 2048 was utilized and acquisition was recorded for 50 frames. Images were acquired for three separate tissue slices for each mouse, with two mouse per group analyzed. Two ROIs were analyzed per image and fluorescent lifetimes were determined by LASX Software. Statistical analysis was conducted via Student’s *t* test (*n* = 12). An outlier test was performed to remove any extraneous data points. Error bars represent standard deviation.

## Data Availability

The data underlying this study are available in the published article and its Supporting Information.

## Supporting Information Available

General eneral experimental procedures; computational calculations; and characterization details including HPLC; HRMS; ^1^H NMR and ^13^C NMR spectra of all compounds (PDF)
