# Supplementary material for: Spatiotemporal Imaging of Catechol Aldehydes in Neural Tissue
Source: JACS Au. 2025 Mar 13;5(4):1717–27. doi: 10.1021/jacsau.4c01249 (PMC12041959; doi:10.1021/jacsau.4c01249)
Supplement: Supplementary file 1 — au4c01249_si_001.pdf [file au4c01249_si_001.pdf]

Spatiotemporal Imaging of Catechol Aldehydes in Neural Tissue

Supporting Information

John M. Talbott,[‡a] Rachel Wills,[‡a] Rajendra Shirke, [‡a] Leslie Hassanein,[b] David Weinshenker,[b] and Monika Raj*[a]

[a]Department of Chemistry, Emory University, Atlanta, GA, 30322, USA.
[b]Department of Human Genetics, Emory University School of Medicine, Atlanta, GA, 30322, USA.
[‡] These authors contributed equally

Table of Contents

**I.**	General	S1
**II.**	Materials	S2
**III.**	Analytical Methods	S2-3
**IV.**	Cell Culture Technique	S3
**V.**	Animal Studies	S3-4
**VI.**	Supplementary Figure 1: Synthesis of **3a**	S4-5
**VII.**	Supplementary Figure 2: Synthesis of **3b**	S6-9
**VIII.**	Supplementary Figure 3: Synthesis of **2a-2d**	S9-13
**IX.**	Supplementary Figure 4: Quantum Yield of **2a** and **2b**	S13-14
**X.**	Supplementary Figure 5: Probes **2a** and **3a** Stability and Chemoselectivity	S14-15
**XI.**	Supplementary Figure 6: Flow Cytometry Cell Death	S15-20
**XII.**	Supplementary Figure 7: Localization of Probe **1a** and **3a** in U-87 MG cells	S21-22
**XIII.**	Supplementary Figure 8: FLIM-FRET Imaging of U-87 MG cells with biological controls	S22-23
**XIV.**	Supplementary Figure 9: FLIM-FRET Imaging of FRET donor **2a** and negative controls **2b**, **2c**, and **2d** with FRET acceptor **3a**	S24-25
**XV.**	Supplementary Figure 10: FLIM-FRET Imaging of U-87 MG cells with exogenous dopamine	S25-26
**XVI.**	Supplementary Figure 11: FLIM-FRET Imaging of U-87 MG cells with exogenous norepinephrine	S26-27
**XVII.**	Supplementary Figure 12: FLIM-FRET Imaging of U-87 MG cells endogenous DOPAL/DOPEGAL	S27-28
**XVIII.**	Supplementary Figure 13: FLIM-FRET Imaging of WT and DBH KO mice	S29
**XIX.**	References	S29
**XX.**	NMR Spectra	S30-36

I. General. All commercial materials (Sigma-Aldrich, Ambeed, and ThermoFisher) were used without further purification. All solvents were reagent or HPLC (Fisher) grade. Percent conversions refer to chromatographically pure compounds. Reaction progress was monitored by TLC plates (TLC Silica gel 60 F_{254}) and visualized with UV lamps.

II. Materials. Rhodamine B, dopamine, and norepinephrine were purchased from Sigma Aldrich. All other small molecules were purchased from CombiBlocks or Ambeed, Inc. with the exception of Daidzin (DDZ) which was purchased from phytolab (89182). AV/PI stains (AV-PacificBlue) were purchased from Biolegend. U-87 MG cells were obtained from the Spangle Lab at Winship Cancer Institute of Emory University School of Medicine.

III. Analytical Methods.

NMR: NMR spectra were recorded on a 400 MHz or 600 MHz Bruker NMR spectrometer. Proton chemical shifts were referenced to residual $CDCl_3$ at 7.26 ppm and carbon chemical shifts were referenced to $CDCl_3$ at 77.16 ppm. Spectra were processed using MestReNova ver. 12.0.4 and TOPSPIN software. The following abbreviations (or combinations thereof) are used to refer to multiplicities: s = singlet, d = doublet, t = triplet, q = quartet, p = quintet, and m = multiplet. Coupling constants (*J*), are reported in Hertz units (Hz).

HPLC: Chemoselectivity reactions were analyzed using high performance liquid chromatography (HPLC) on an Agilent 1100 series equipped with a 5 µm particle size, C-18 reversed-phase column. All separations involved a mobile phase of water with 0.1% formic acid (solvent A) and acetonitrile with 0.1% formic acid (solvent B). The HPLC method employed a linear gradient of 0-60% solvent B over 30 minutes at ambient temperature with a flow rate of 1 mL/min. The eluent was monitored by absorbance at 220 nm and 280 nm.

HRMS. High resolution MS data were acquired on Thermo Exactive Plus using a heated electrospray source. The solution was infused at a rate of 10-25 µL min^{-1} electrospray using 3.3 kV. The typical settings were Capillary temp 320 °C. S-lens RF level was between 30-80 with an AGC setting of 1 E6. The maximum injection time was set to 50 ms. Spectra were taken at 140,000 resolutions at m/z 200 using Tune software and analyzed with ThermoFischer's Freestyle software. ver. 1.8.63.0.

Fluorimeter: Fluorescence spectroscopy was performed with an Agilent Cary Eclipse. Probe **1a** had an excitation of 488 nm and an emission of 507 nm. Probe **3a** has excitation of 566 nm and an emission of 602 nm.

Microscopy: Confocal Imaging with fluorescence lifetime imaging microscopy (FLIM) was performed on a Stellaris® 8 Leica DMi8 microscope.

Cells were plated in an IBIDI 8-well glass bottom chamber at a density of 25,000 cells per well in media and allowed to adhere overnight at 37 °C, 5% CO_2. The media was aspirated and samples pre-dissolved in DMSO were introduced into each well at the respective concentrations. Incubation was performed at 37 °C, 5% CO_2.

Cells were imaged live using an incubator-equipped Leica Stellaris® 8 microscope (20x objective) with fast lifetime contrast (FALCON) module. Samples were excited using an 80 MHz pulsed white light laser tuned to 488 nm for both intensity and fluorescence

lifetime measurements. Emitted photons were detected using HyD® X (GaAsP hybrid photocathode).

Fluorescence lifetime imaging microscopy (FLIM) was conducted with living cells. Image resolution of 1024 x 1024 was utilized. Acquisition was recorded for 50 frames. 5 pictures were acquired for each well. Probe **2a** exhibited three fluorescent lifetimes representing three distinct exponential components for the probe. This was accounted for in all FLIM analysis.

Fluorescent lifetimes were determined by LASX Software N-exponential reconvolution formula of **2a** (donor) alone as seen with formula (1) where n = number of exponential components, A = amplitudes (Exponential pre-factors), τ = exponential decay time (lifetimes), $Bkgr$ = tail offset (correction for background such as afterpulsing, dark counts, and environmental light, $Shift_{IRF}$ = IRF Shift (correction for IRF displacement such as wavelength dependent zero time of the detector), $Bkgr_{IRF}$ = IRF Background (correction for IRF background).

$$y(t) = \{IRF(t + Shift_{IRF}) + Bkgr_{IRF}\} \otimes \left\{\sum_{i=0}^{n-1} A[i]e^{\left(-\frac{t}{\tau[i]}\right)} + Bkgr\right\} \quad (1)$$

The muti-exponential donor equation (also equation 1) can be used to find the fluorescent lifetime of all other images, with the added calculation of FRET efficiency (E_{FRET}) by inputting the average donor lifetime (τ_D) into equation (2) where τ_{AvAmp} = mean decay time of the image.

$$E_{FRET} = \left(1 - \frac{\tau_{AvAmp}}{\tau_D}\right) \quad (2)$$

Pixel analysis was conducted by excluding any pixels with a photon count less than 10% of the maximum photon count to remove additional background noise. Mean fluorescence lifetime weighted by amplitude was recorded for all analysis. All χ^2 values for lifetime decay curves were less than 2.5.

IV. Cell Culture Technique: Cells were maintained at 37 °C and 5% CO_2. U-87 MG cells were obtained from the Spangle lab at Winship Cancer Institute of Emory University. U-87 MG cells were cultured in DMEM/F12 (1:1) supplemented with 10% (V/V) fetal bovine serum (FBS) and 1% (V/V) penicillin/streptomycin (100 µg/mL).

V. Animal Studies:

Dopamine beta-hydroxylase knockout (*Dbh -/-)* mice, maintained on a mixed 129/SvEv and C57BL/6 J background as previously described,[1,2] were used in this study. Heterozygous (*Dbh* +/−) littermates were used as controls because their behavior and catecholamine levels are indistinguishable from wild-type (*Dbh* +/+) mice.[2-4] Animals were maintained on a 12:12 light:dark cycle (lights on at 0700), and food and water were *ad Libitum*. All experiments were conducted at Emory University in accordance with the National Institutes of Health Guideline for the Care and Use of Laboratory Animals and approved by the Emory Institutional Animal Care and Use Committee.

Mice were anesthetized with isoflurane and euthanized by rapid decapitation. Mouse brains were rapidly dissected on ice and flash-frozen in isopentane on dry ice. Samples were stored at -20 °C for 24 hours. Brains were transferred to 5 mL centrifuge tubes containing 10 μM probe **1a** in PBS (1.5 μL of 10 mM stock of probe **1a** in DMSO into 1498.5 μL of PBS) and allowed to diffuse for 16 hours in a 4 °C refrigerator. Solution was removed and replaced with 20 μM probe **3a** in PBS (3 μL of 10 mM stock of probe **3a** in DMSO into 1497 μL of PBS) and allowed to diffuse for 8 hours in a 4 °C refrigerator. Solution was removed and brains were fixed with paraformaldehyde for 8 hours. Brains were embedded in OT medium (Tissue-Tek) and sectioned by cryostat into 60 μM thick coronal sections at the level of the LC or SN. Sections were immediately transferred to glass Superfrost Plus slides which were then coverslipped with Fluoromount-G (Southern Biotech, Birmingham, AL) and allowed to dry before imaging.

VI. Supplementary Figure 1: Synthesis of Probe 3a

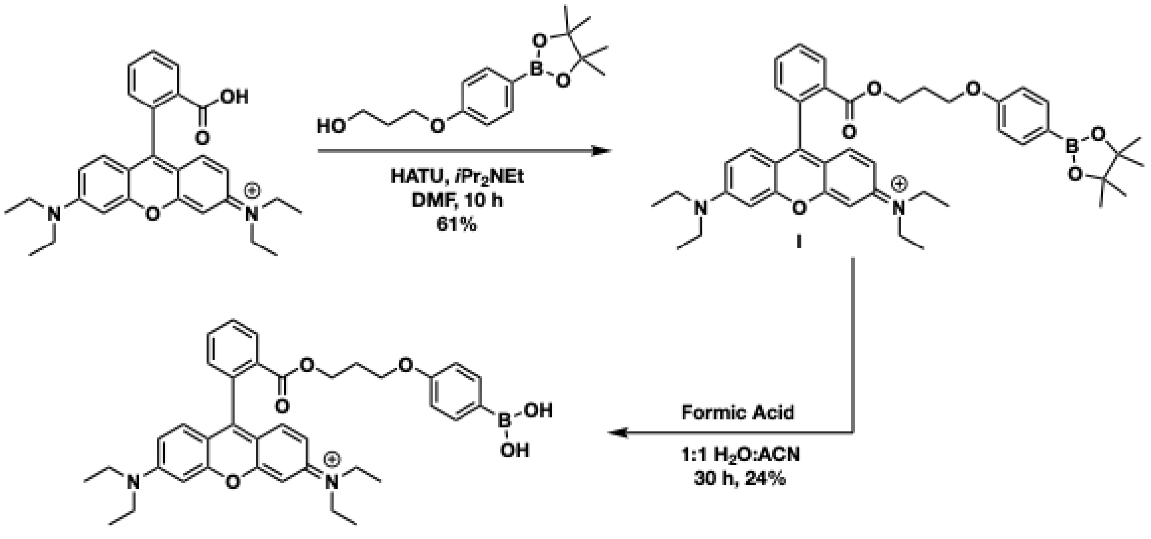

Representative procedure for the synthesis of N-(6-(Diethylamino)-9-(2-((3-(4-(4,4,5,5-tetramethyl-1,3,2-dioxaborolan-2-yl)phenoxy)propoxy)carbonyl)phenyl)-3H-xanthen-3-ylidene)-N-ethylethanaminium (Figure S1, I):

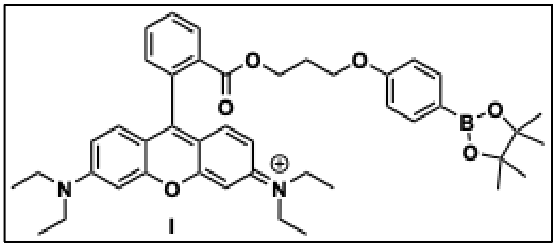

An oven-dried 25 mL RBF was charged with Rhodamine B (1.0 equiv.), HATU (2.0 equiv.), *i*Pr$_2$NEt (2.0 equiv.) in dry DMF at RT. After 15 minutes, Boronate ester (1.3 equiv.) was added and stirred at RT for 10 h. The reaction progress was monitored by TLC. Upon completion, the reaction mixture was dissolved in DCM. The organic layer was washed with water and separated. The aqueous layer was back extracted with DCM. The combined organic layers were washed with saturated aqueous NaCl, dried over $MgSO_4$, and concentrated under reduced pressure. The

crude residue was purified by silica gel column chromatography using DCM/MeOH (9:1) as eluent to afford coupled product. This compound was isolated as pink solid with 61% yield.
R*f* = 0.4 (9:1 = DCM/MeOH).

^{1}H NMR (400 MHz, $CDCl_3$): δ 8.32 (dd, *J* = 8.0, 1.3 Hz, 1H), 7.82 (td, *J* = 7.5, 1.3 Hz, 1H), 7.75 (td, *J* = 7.8, 1.6 Hz, 1H), 7.73 – 7.69 (m, 1H), 7.34 – 7.29 (m, 1H), 7.27 (d, *J* = 14.6 Hz, 1H), 7.08 (dd, *J* = 9.6, 7.7 Hz, 2H), 6.98 – 6.87 (m, 1H), 6.82 (h, *J* = 8.6, 8.2 Hz, 4H), 6.76 (d, *J* = 8.2 Hz, 1H), 4.25 (td, *J* = 6.2, 1.9 Hz, 2H), 3.85 (q, *J* = 6.2 Hz, 2H), 3.59 (q, *J* = 7.1 Hz, 8H), 1.99 – 1.90 (m, 2H), 1.34 (s, 12H), 1.32 (t, *J* = 7.2 Hz, 12H).

^{13}C NMR (101 MHz, $CDCl_3$): δ 165.09, 162.58, 161.15, 158.79, 157.72, 157.70, 155.52, 136.50, 136.46, 133.44, 133.11, 131.41, 131.23, 130.41, 130.25, 114.12, 113.69, 113.46, 96.36, 83.62, 64.03, 62.53, 46.03, 36.53, 31.44, 28.33, 24.86, 24.83, 12.53.

HRMS (ESI): *m/z* $[M]^+$calcd. for $[C_{43}H_{52}BN_2O_6]^+$ 703.3913, found: 703.3910.

Representative procedure for the synthesis of N-(9-(2-((3-(4-boronophenoxy)propoxy)carbonyl)phenyl)-6-(diethylamino)-3H-xanthen-3-ylidene)-N-ethylethanaminium (Figure S1, 3a):

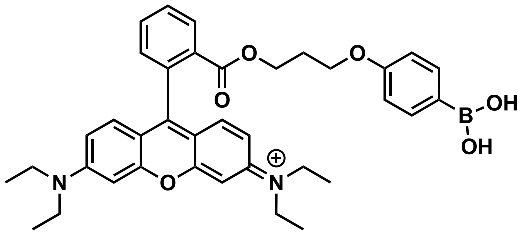

3a

The dark pink Rhodium B adduct (1.0 equiv.) was dissolved in ACN:H_2O (1:1) followed by the addition of formic acid (10 equiv.) at RT and stirred for 30 h. The reaction progress was monitored by TLC. Upon completion, the reaction mixture was dissolved in DCM. The organic layer was washed with water and separated. The aqueous layer was back extracted with DCM. The combined organic layers were washed with saturated aqueous NaCl, dried over $MgSO_4$, and concentrated under reduced pressure. The crude residue was purified by silica gel column chromatography using DCM/MeOH (9:1) as eluent to afford **3a**. This compound was isolated as pink solid with 24% yield.

R*f* = 0.4 (9:1 = DCM/MeOH).

^{1}H NMR (400 MHz, $CDCl_3$): δ 8.38 (dd, *J* = 7.4, 1.9 Hz, 1H), 8.11 (d, *J* = 8.4 Hz, 1H), 7.90 (d, *J* = 8.4 Hz, 1H), 7.79 (td, *J* = 7.0, 1.7 Hz, 2H), 7.75 (d, *J* = 8.2 Hz, 2H), 7.61 (t, *J* = 7.6 Hz, 1H), 7.49 (t, *J* = 7.7 Hz, 1H), 6.91 (d, *J* = 9.5 Hz, 1H), 6.74 – 6.72 (m, 1H), 6.68 (d, *J* = 2.4 Hz, 1H), 6.51 (d, *J* = 8.3 Hz, 1H), 6.39 (s, 1H), 4.27 (t, *J* = 5.5 Hz, 2H), 4.15 – 4.08 (m, 1H), 3.86 (dd, *J* = 7.2, 5.0 Hz, 1H), 3.69 (t, *J* = 6.6 Hz, 2H), 3.64 – 3.52 (m, 8H), 1.30 (t, *J* = 7.0 Hz, 12H).

^{13}C NMR (101 MHz, $CDCl_3$): δ 165.2, 162.8, 159.9, 158.4, 157.5, 155.4, 143.5, 136.0, 133.4, 132.9, 131.6, 131.1, 130.3, 130.2, 128.9, 17.8, 125.2, 120.33, 119.4, 114.1, 113.2, 113.0, 109.3, 104.8, 96.2, 64.2, 62.6, 46.1, 28.5, 24.8 24.8, 12.6.

HRMS (ESI): *m/z* $[M]^+$calcd. for $[C_{37}H_{42}BN_2O_6]^+$: 621.3136, found: 621.3135.

VII. Supplementary Figure 2: Synthesis of 3b

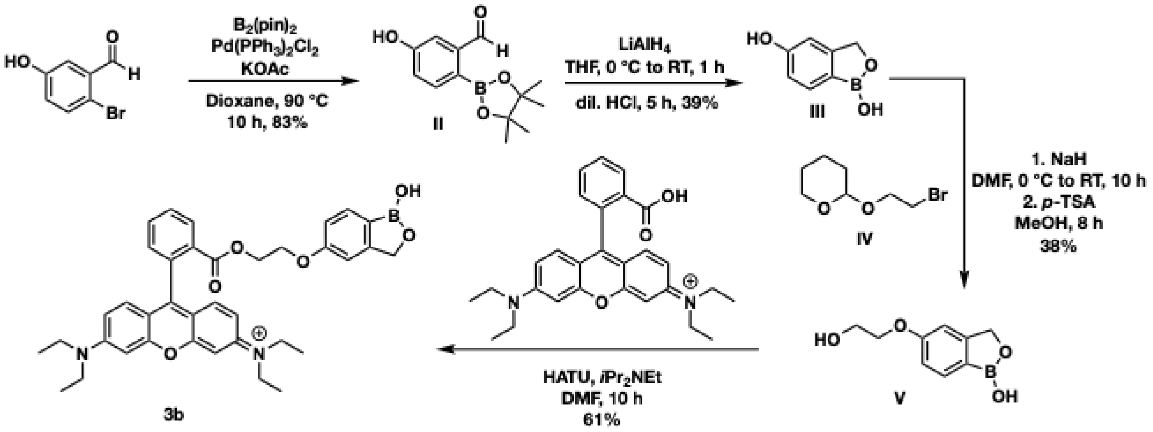

Representative procedure for the synthesis of 5-hydroxy-2-(4,4,5,5-tetramethyl-1,3,2-dioxaborolan-2-yl)benzaldehyde (Figure S2, II):

Procedure adapted from Glorius and coworkers.[5]

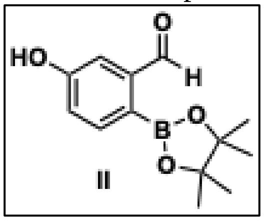

2-Bromo-5-hydroxybenzaldehyde (1.0 equiv), $PdCl_2(PPh_3)_2$ (10 mol%), $B_2(pin)_2$ (1.3 equiv), and KOAc (3.0 equiv) were charged to a RBF. Dry 1,4-dioxane was added to the RBF. The mixture was degassed then stirred at 90 °C for 10 h. Upon completion, the reaction mixture was quenched with dil. HCl and extracted with ethyl acetate. The combined organic layers were washed with saturated aqueous NaCl, dried over $MgSO_4$, and concentrated under reduced pressure. The residue was purified by silica gel column chromatography using hexane/ethyl acetate (5:2) as eluent to afford coupled product, 5-hydroxy-2-(4,4,5,5-tetramethyl-1,3,2-dioxaborolan-2-yl)benzaldehyde. This compound was isolated as colorless thick oil with 83% yield.

$\mathbf{R}_f$ = 0.4 (9:1 = Hexane/EtOAc).

^{1}H NMR (400 MHz, $CDCl_3$): δ 10.56 (s, 1H), 8.15 – 7.91 (m, 1H), 7.77 (d, *J* = 8.2 Hz, 1H), 7.49 (d, *J* = 2.6 Hz, 1H), 7.06 (dd, *J* = 8.2, 2.5 Hz, 1H), 1.32 (s, 12H). Matches literature spectra.[1]

^{13}C NMR (101 MHz, $CDCl_3$): δ 196.11, 159.05, 143.13, 138.22, 120.99, 113.84, 84.32, 24.81.

HRMS (ESI): *m/z* $[M]^+$calcd. for $[C_{13}H_{17}BO_4]^+$ = 248.1220, found 248.1221.

Representative procedure for the synthesis of Benzo[c][1,2]oxaborole-1,5(3H)-diol (Figure S2, III):

Procedure adapted from Glorius and coworkers.[5]

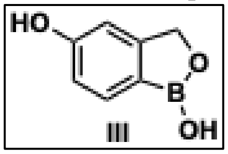

To a RBF at 0 °C, 5-hydroxy-2-(4,4,5,5-tetramethyl-1,3,2-dioxaborolan-2-yl)benzaldehyde (1 equiv.) was added then dissolved in THF. $LiAlH_4$ (2.0 equiv.) was added portion wise over 5 minutes to the reaction mixture. The reaction mixture was stirred for 1 h at 0 °C. Upon completion, the reaction mixture was quenched with dilute aqueous HCl and stirred for 5 h. Upon completion, the reaction mixture was poured into water. The organic layer was extracted with ethyl acetate and washed with saturated aqueous NaCl. $MgSO_4$ was added to the organic fraction and stirred for 10 minutes before the heterogeneous mixture was filtered and filtrate concentrated in vacuo. The residue was purified by silica gel column chromatography using hexane/ethyl acetate (1:1) as eluent to afford benzoxaborole product Benzo[c][1,2]oxaborole-1,5(3H)-diol. This compound was isolated as a thick colorless oil with 39% yield.

R_f = 0.4 (1:1 = Hexane/EtOAc).

^{1}H NMR (400 MHz, DMSO-d_6): δ 9.76 (s, 1H), 8.88 (s, 1H), 7.53 (d, J = 8.6 Hz, 1H), 6.79 – 6.69 (m, 2H), 4.87 (s, 2H). Matches literature spectra.[5]

Representative procedure for the synthesis of 2-(2-bromoethoxy)tetrahydro-2H-pyran (Figure S2, IV):

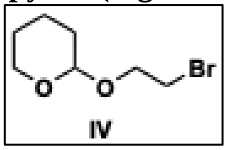

To a stirred solution of 3,4-dihydro-2H-pyran (3 equiv.) and *p*-toluenesulfonic acid monohydrate (10 mol%) at 0 °C, 2-bromoethanol (1 equiv.) was added over a 10-minute period. The resulting solution was gradually warmed to room temperature and stirred for 20 h. Upon completion, the reaction mixture was quenched with a saturated aqueous solution of $NaHCO_3$ and extracted twice with EtOAc. The combined organic extracts were dried over anhydrous Na_2SO_4, filtered, and solvent removed in vacuo. The crude isolate was purified by fractional distillation under vacuum to isolate the product in 68% yield.

R_f = 0.6 (1:3 = Hexane/EtOAc).

^{1}H NMR (400 MHz, DMSO-d_6): δ 4.68 (t, J = 3.6 Hz, 1H), 4.02 (dt, J = 11.2, 6.2 Hz, 1H), 3.90 (ddd, J = 11.2, 8.3, 3.3 Hz, 1H), 3.78 (dt, J = 11.2, 6.4 Hz, 1H), 3.57 – 3.49 (m, 3H), 1.85 (dd, J = 9.7, 3.7 Hz, 1H), 1.78 – 1.70 (m, 1H), 1.68 – 1.50 (m, 4H).

^{13}C NMR (101 MHz, DMSO-d_6): δ 98.93, 67.53, 62.27, 30.86, 30.43, 25.36, 19.26.

HRMS (ESI): *m/z* $[M]^+$calcd. for $[C_7H_{13}BrO_2]^+$ = 208.0099, found 208.0101.

Representative procedure for the synthesis of 5-(2-hydroxyethoxy)benzo[c][1,2]oxaborol-1(3H)-ol (Figure S2, V):

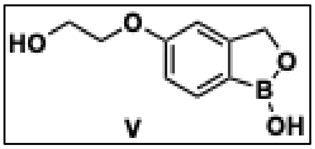

1) A RBF was charged with benzoxaborole adduct, Benzo[c][1,2]oxaborole-1,5(3H)-diol (1 equiv.) in DMF at 0 °C. NaH (1.3 equiv.) was added portion wise over 5 minutes to the reaction mixture. After 1 h, 2-(2-bromoethoxy)tetrahydro- 2H-pyran (1 equiv.) was added and the reaction mixture was stirred for 10 h at RT. Upon completion, the reaction mixture was quenched with dil. aqueous NH_4Cl and extracted with ethyl acetate. The combined organic layers were washed with saturated aqueous NaCl, dried over $MgSO_4$, and concentrated under reduced pressure.
2) The crude reaction mixture was taken in MeOH followed by the addition of *p*-TSA (0.3 equiv) and stirred for 8 h at RT. Upon completion, the MeOH was removed from reaction mixture by reduced pressure. The organic layer was extracted with ethyl acetate and washed with saturated aqueous NaCl. The combined organic layers were washed with saturated aqueous NaCl, dried over $MgSO_4$, and concentrated under reduced pressure. The residue was purified by silica gel column chromatography using hexane/ethyl acetate (1:1) as eluent to afford 5-(2-hydroxyethoxy)benzo[c][1,2]oxaborol-1(3H)-ol. This compound was isolated as a thick colorless oil with 38% yield.

R_f = 0.3 (1:1 = Hexane/EtOAc).

^{1}H NMR (400 MHz, DMSO-*d*$_6$): δ 8.99 (s, 1H), 7.61 (d, *J* = 8.1 Hz, 1H), 6.99 – 6.95 (m, 1H), 6.91 (dd, *J* = 8.1, 2.2 Hz, 1H), 4.92 (s, 2H), 4.89 (t, *J* = 5.5 Hz, 1H), 4.02 (t, *J* = 5.0 Hz, 2H), 3.76 – 3.69 (m, 2H).

^{13}C NMR (101 MHz, DMSO-*d*$_6$): δ 161.61, 156.80, 132.33, 115.14, 106.74, 70.10, 69.97, 59.93.

HRMS (ESI): *m/z* $[M]^+$calcd. for $[C_9H_{11}BO_4]^+$ 194.0750, found: 194.0749.

Representative procedure for the synthesis of N-(6-(diethylamino)-9-(2-((2-((1-hydroxy-1,3-dihydrobenzo[c][1,2]oxaborol-5-yl)oxy)ethoxy)carbonyl)phenyl)-3H-xanthen-3-ylidene)-N-ethylethanaminium (3b):

An oven-dried 25 mL RBF was charged with Rhodamine B (1.0 equiv) in dry DMF at RT.

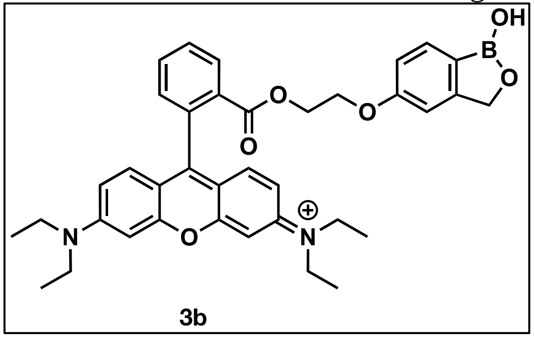

Then, HATU (2.0 equiv), DIPEA (2.0 equiv) and protected benzoxaborole, 5-(2-hydroxyethoxy)benzo[c][1,2]oxaborol-1(3H)-ol (1.3 equiv) were added and the reaction mixture was stirred for 10 h. The reaction progress was monitored by TLC. Upon completion, the reaction mixture was diluted in DCM. The organic layer was washed with water and separated; the aqueous layer was extracted with DCM. The combined organic

layers were washed with saturated aqueous NaCl, dried over $MgSO_4$, and concentrated under reduced pressure. The crude residue was purified by silica gel column chromatography using DCM/MeOH (9:1) as eluent to afford coupled product, **3b**. This compound was isolated as pink solid with 61%.

R*f* = 0.4 (9:1 = DCM/MeOH).

^{1}H NMR (400 MHz, $CDCl_3$): δ 8.31 (d, *J* = 7.9 Hz, 1H), 8.03 (s, 1H), 7.79 (t, *J* = 7.5 Hz, 1H), 7.73 (t, *J* = 7.7 Hz, 1H), 7.69 (d, *J* = 8.1 Hz, 1H), 7.27 (d, *J* = 7.4 Hz, 1H), 7.05 (d, *J* = 9.3 Hz, 2H), 6.78 (dd, *J* = 9.6, 2.5 Hz, 2H), 6.70 (d, *J* = 2.2 Hz, 1H), 6.62 (d, *J* = 2.5 Hz, 2H), 4.99 (s, 2H), 4.35 – 4.30 (m, 2H), 3.82 (t, *J* = 4.2 Hz, 2H), 3.55 – 3.50 (m, 8H), 1.27 (t, *J* = 7.0 Hz, 12H).

^{13}C NMR (101 MHz, $CDCl_3$): δ 165.21, 162.85, 161.01, 158.28, 157.63, 157.59, 156.39, 155.41, 133.27, 133.11, 131.83, 131.65, 131.63, 131.18, 130.35, 130.14, 129.84, 115.17, 114.08, 113.49, 105.81, 96.28, 70.75, 65.57, 63.97, 46.00, 12.51.

HRMS (ESI): *m/z* $[M]^+$calcd. for $[C_{37}H_{40}BN_2O_6]^+$ 619.2974, found: 619.2975.

VIII. Supplementary Figure 3: Synthesis of 2a-2d

Representative synthesis of Diethyl 10-(2-(3,4-dihydroxybenzyl)-1*H*-benzo[*d*]imidazol-5-yl)-5,5-difluoro-1,3,7,9-tetramethyl-5*H*-4λ^4,5λ^4-dipyrrolo[1,2-*c*:2',1'-*f*][1,3,2]diazaborinine-2,8-dicarboxylate (2a)

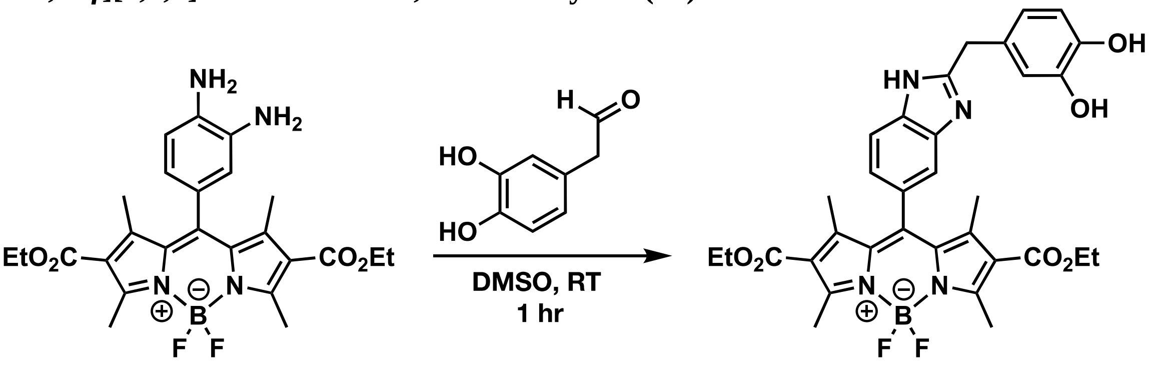

1a 2a

To a stirred solution of **1a** (1 equiv.) in DMSO, DOPAL (10 equiv.) was added, and the reaction was stirred at RT for 1 h. Reaction was purified by HPLC then frozen and lyophilized revealing a dark orange solid **2a**. Product was kept in -80 °C away from light and split into 10 mM batches in DMSO when utilized for subsequent experiments.

HPLC: 0-60% solvent B over 30 minutes. Retention time = 26.1 minutes
HRMS (ESI): *m/z* $[M+H^+]$ calcd. for $[C_{33}H_{34}BF_2N_4O_6]^+$ = 631.2534, found 631.2542.

HPLC Trace of 1b

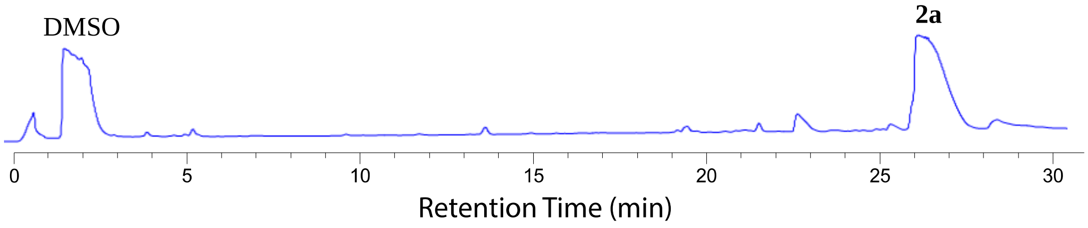

HRMS of 1b

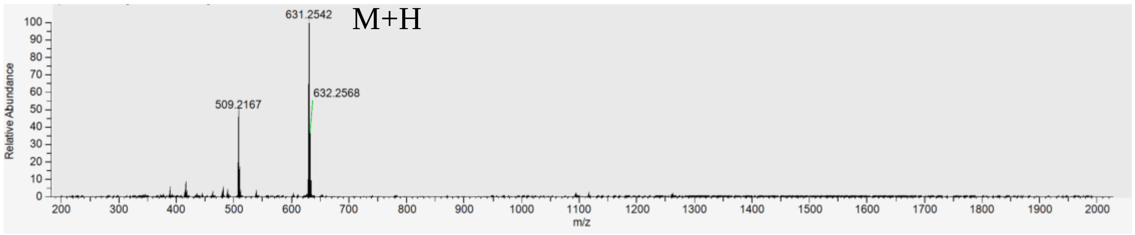

Representative synthesis of Diethyl 10-(2-ethyl-1*H*-benzo[*d*]imidazol-5-yl)-5,5-difluoro-1,3,7,9-tetramethyl-5*H*-4λ4,5λ4-dipyrrolo[1,2-*c*:2',1'-*f*][1,3,2]diazaborinine-2,8-dicarboxylate (2b)

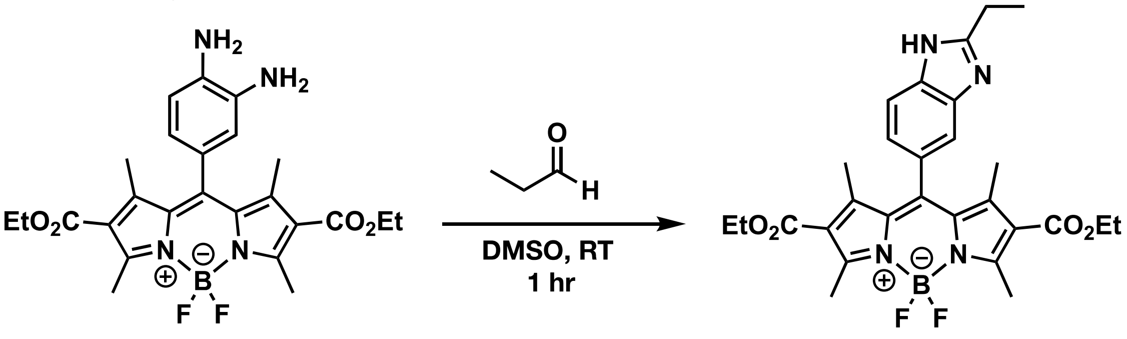

1a **2b**

To a stirred solution of **1a** (0.01 mmol, 1 equiv.) in DMSO (1 mL), propanal (0.1 mmol, 10 equiv.) was added, and the reaction was stirred at RT for 1 h. Reaction was purified by HPLC then frozen and lyophilized revealing a dark orange solid **2b**. Product was kept in -80 °C away from light and split into 10 mM batches in DMSO when utilized for subsequent experiments.

HPLC: 20-100% solvent B over 30 minutes. Retention time = 16.9 minutes
HRMS (ESI): *m/z* [M+H$^+$] calcd. for $[C_{28}H_{32}BF_2N_4O_4]^+$ = 537.2479, found 537.2482.
Matches literature spectra.[2]

HPLC Trace of 2b

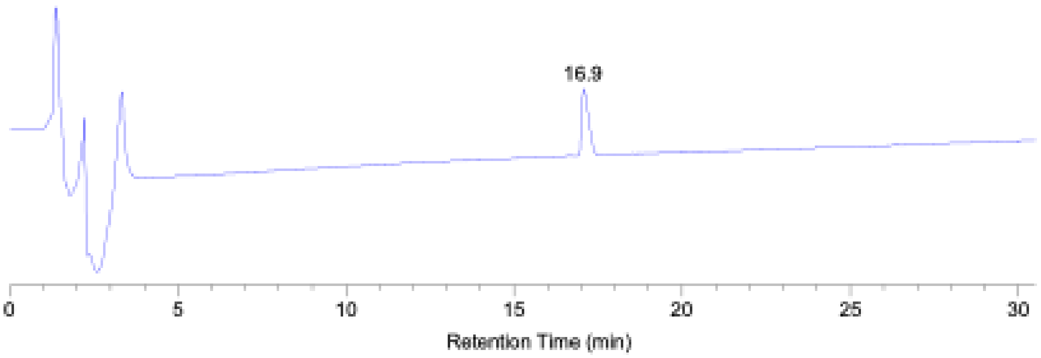

HRMS of 2b

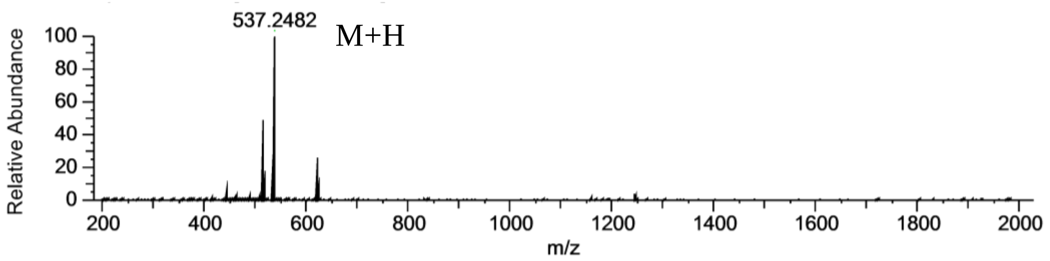

Representative synthesis of 5-(2,8-bis(ethoxycarbonyl)-5,5-difluoro-1,3,7,9-tetramethyl-5H-4λ4,5λ4-dipyrrolo[1,2-c:2',1'-f][1,3,2]diazaborinin-10-yl)-2-nonyl-1H-benzo[d]imidazol-3-ium (2c)

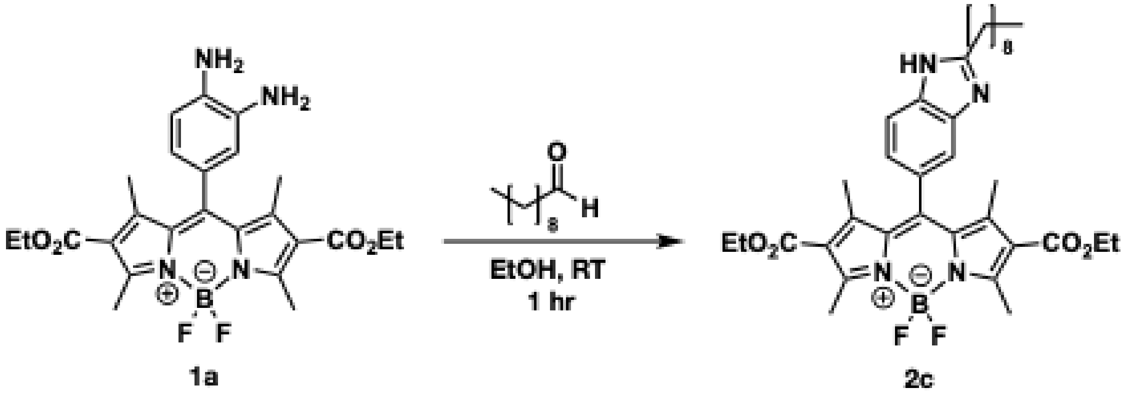

To a stirred solution of **1a** (0.01 mmol, 1 equiv.) in EtOH (1 mL), decanal (0.2 mmol, 20 equiv.) was added, and the reaction was stirred at RT for 1 h. Reaction was purified by HPLC then frozen and lyophilized revealing a dark orange solid **2c**. Product was kept in -80 °C away from light and split into 10 mM batches in DMSO when utilized for subsequent experiments.

HPLC: 20-100% solvent B over 30 minutes. Retention time = 23.8 minutes
HRMS (ESI): *m/z* [M+H$^+$] calcd. for $[C_{35}H_{46}BF_2N_4O_4]^+$ = 635.3575, found 635.3577.

HPLC Trace of 2c

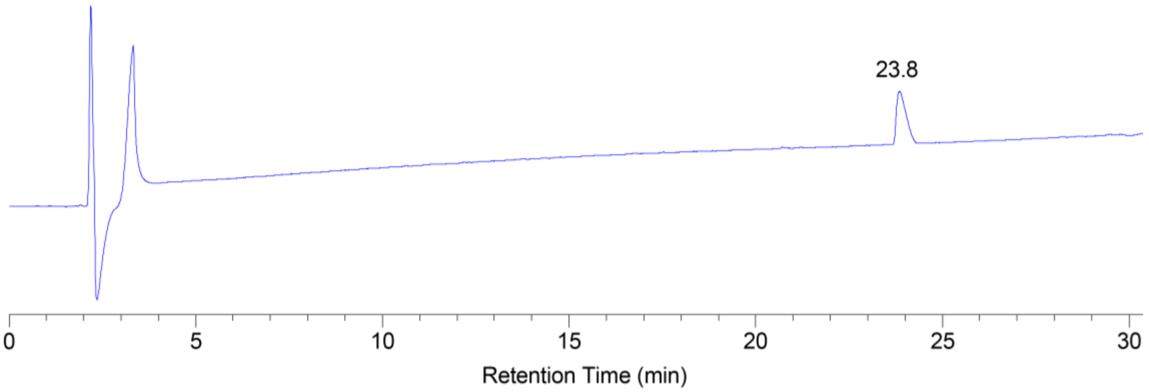

HRMS of 2c

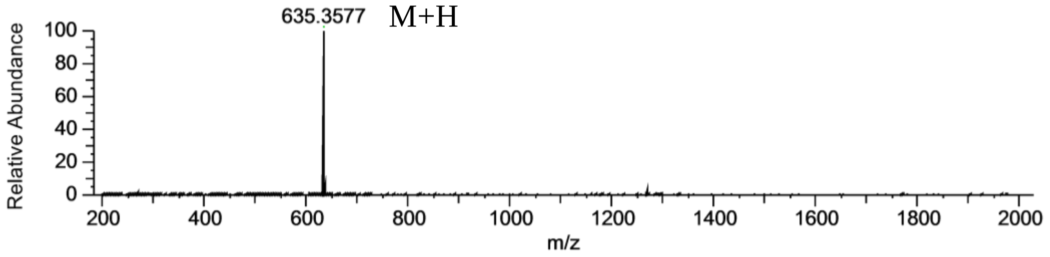

Representative synthesis of Diethyl 10-(2-(3,4-dihydroxybenzyl)-1*H*-benzo[*d*]imidazol-5-yl)-5,5-difluoro-1,3,7,9-tetramethyl-5*H*-4λ[4],5λ[4]-dipyrrolo[1,2-*c*:2',1'-*f*][1,3,2]diazaborinine-2,8-dicarboxylate (2d)

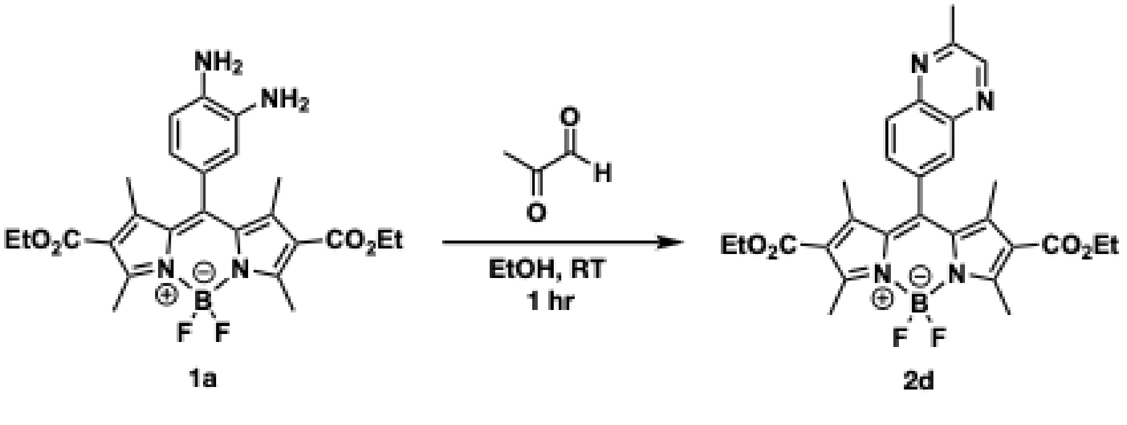

To a stirred solution of **1a** (0.01 mmol, 1 equiv.) in EtOH (1 mL), MGO (0.2 mmol, 10 equiv.) was added, and the reaction was stirred at RT for 1 h. Reaction was purified by HPLC then frozen and lyophilized, revealing a dark orange solid **2d**. Product was kept in -80 °C away from light and split into 10 mM batches in DMSO when utilized for subsequent experiments.

HPLC: 20-100% solvent B over 30 minutes. Retention time = 22.2 minutes
HRMS (ESI): *m/z* [M+H$^+$]calcd. for $[C_{28}H_{30}BF_2N_4O_4]^+$ = 535.2323, found 535.2328.

HPLC Trace of 2d

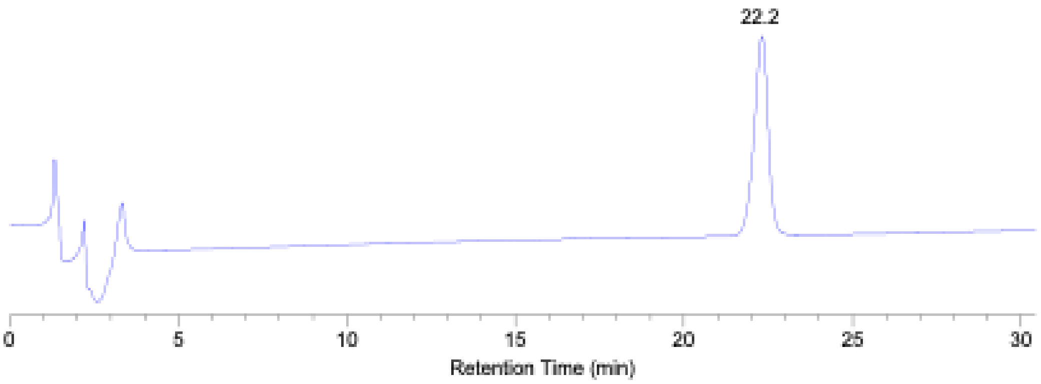

HRMS of 2d

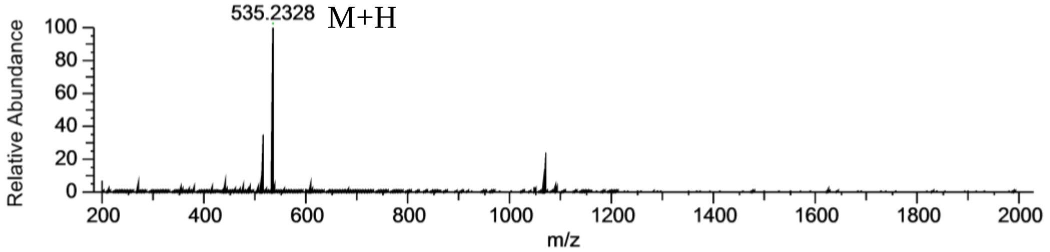

IX. Supplementary Figure 4: Quantum yield determination of probe 2a and 2b. Quantum yield was calculated using the area under the curve of fluorescence versus absorption. Absorption was measured with a Cary 3500 UV-Vis utilizing the samples at using four separate concentrations with values being divided by ten for accurate analysis. Fluorescence area was measured with a Cary Eclipse fluorimeter using the same samples above with a 10X dilution. All measurements were run in triplicate. Quantum yields of probe **2a** and **2b** were determined using Cy2 as a reference compound. The following equation was used to calculate quantum yield:

$$\Phi = \Phi_r \times \frac{m}{m_r} \times (\frac{n}{n_r})^2$$

Φ is the quantum yield; m is the slope of the line described above; n is the refractive index of the solvent. Subscript r denotes the appropriate values for the reference (Cy2).

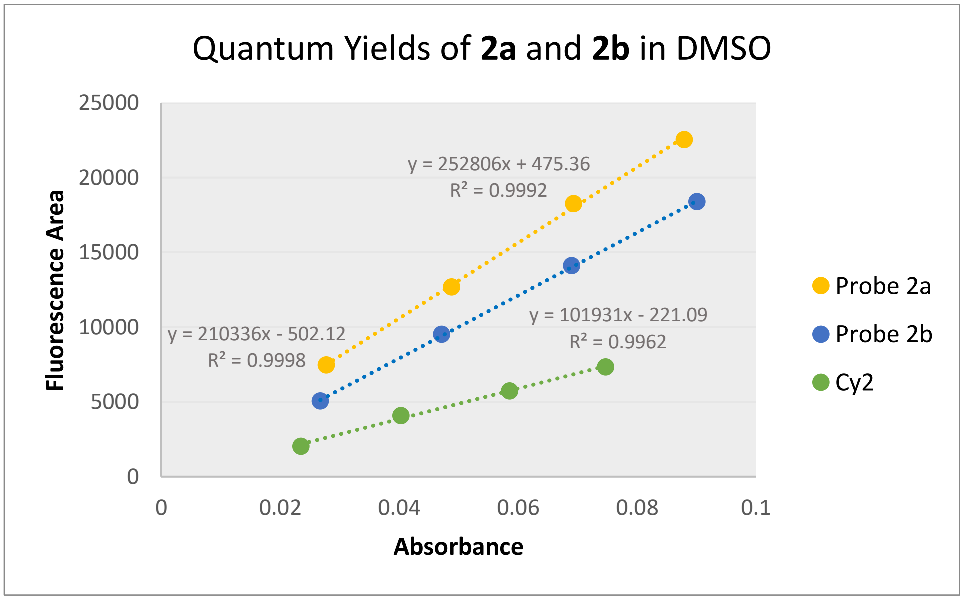

X: Supplementary Figure 5: Probes 2a and 3a Stability, Kinetics, and Chemoselectivity

Absorbance and Emission of 2a and 3a

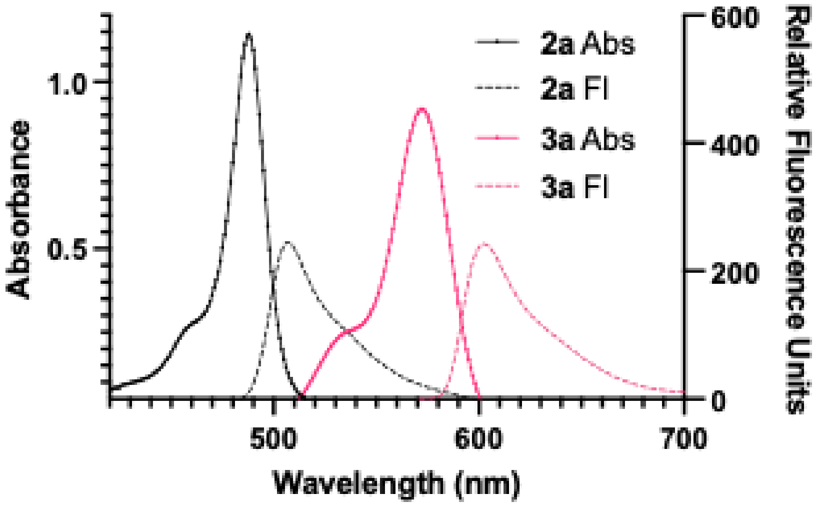

FRET Stability

Probe **2a** (1 equiv.) was incubated with Probe **3a** (20 equiv.) in phenol-red free Gibco RPMI 1640 media containing 10% FBS and fluorescence intensity was measured at set time points. This experiment was repeated in triplicate.

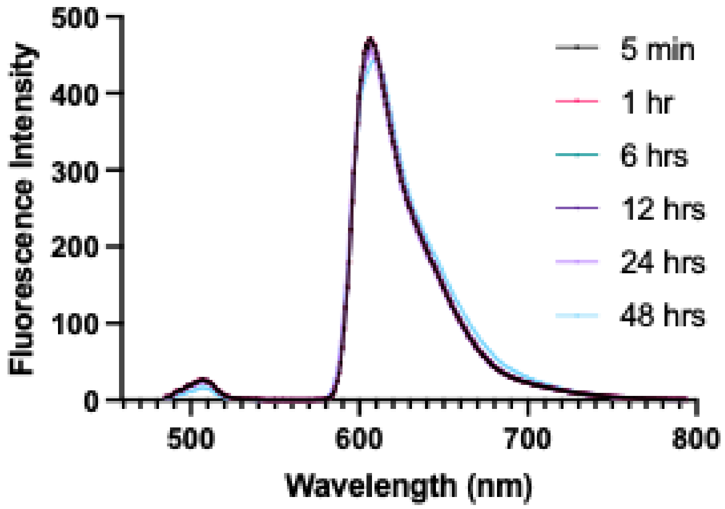

FRET Chemoselectivity:
Probe **1a** (1 equiv.) was incubated with 100 eq. of each metabolite and 20 equiv. **3a** in DMSO for 2 hours, then fluorescence intensity was recorded. This was repeated in triplicate.

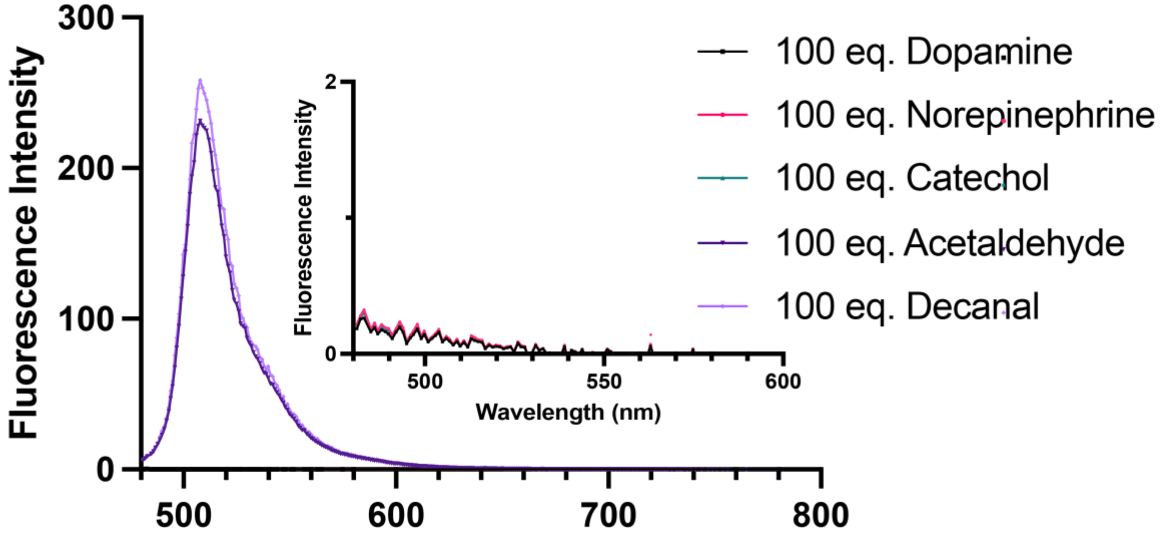

XI: Supplementary Figure 6: Flow Cytometry Cell Death

Flow Cytometry: Cells were grown in 60 x 15 mm Nunclon™ dishes. Stock solutions of probe **1a, 3a,** and **3b** and drugs dopamine (DA), norepinephrine (NE), dexamethasone (Dexa), benomyl (Ben), and Diadzin (DDZ) or combinations thereof were prepared in DMSO before being diluted to the final desired concentration in 4 mL of media. Cells were placed in the incubator for treatment for 2 hours. Cells were then detached with trypsin and stained using Annexin V/PI following the manufacturer's protocol. To avoid fluorescent crosstalk, commercially available Annexin V (AV) conjugated to Pacific Blue (PB) was used to determined apoptosis. Propidium Iodide (PI) was used to determine necrosis within the cellular populations. Cells were analyzed via flow cytometry within 1

hour to quantify cell death. A V470/15 laser was used to detect AV-PB (apoptosis) while a B-710/50 laser was used to detect PI (necrosis). FlowJo software was used to analyze cytometry data. Gating was applied based on AV/PI/Unstained control samples run for each batch of samples; gating slightly varied between samples as seen in representative graphs below. All compounds were analyzed in triplicate and were normalized to naïve cell viability (in triplicate). The dashed line represents 85% cell viability.

Cell viability < 85% was observed for 30-40 µM **3a** and 20-40 µM **3b**. Cell viability approaching 15% was observed for the combination of 10 µM **1a**, 20 µM **3a**, 50 µM Ben, 50 µM Dexa, and either 1 mM of DA or NE (All + DA and All + NE). This follows observations during microscopy that cells dosed with these highly elevated systems begin significantly dying after ~4 hours (2 hours of dosing + 2 hours of imaging).

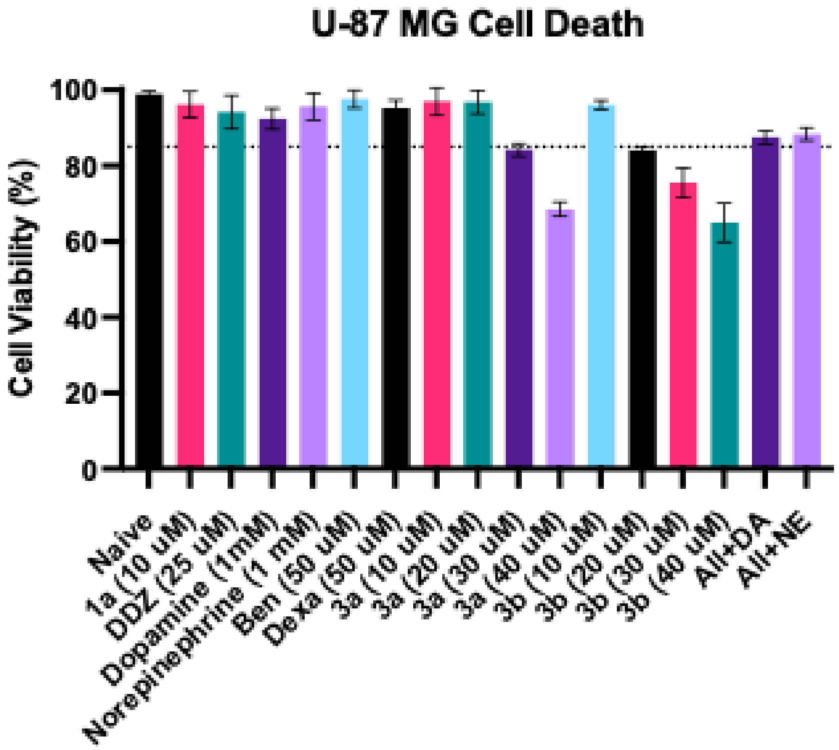

Representative Flow Cytometry for Each Compound with U-87 MG Cells

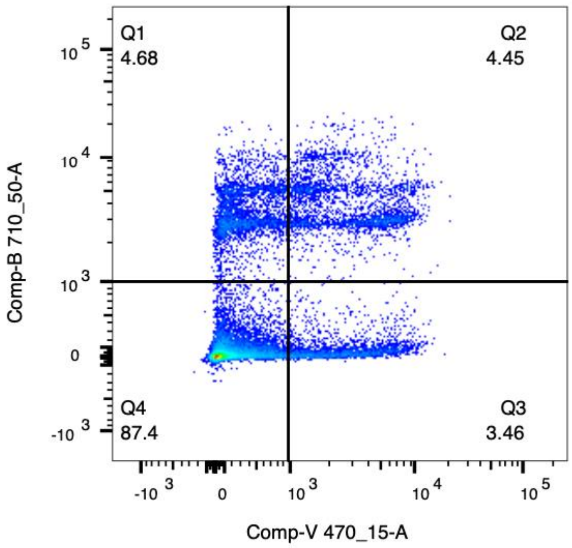

Naïve Cells

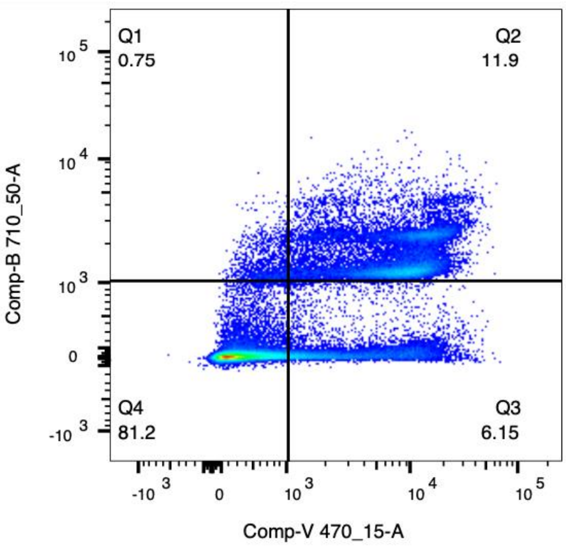

10 µM Probe **1a**

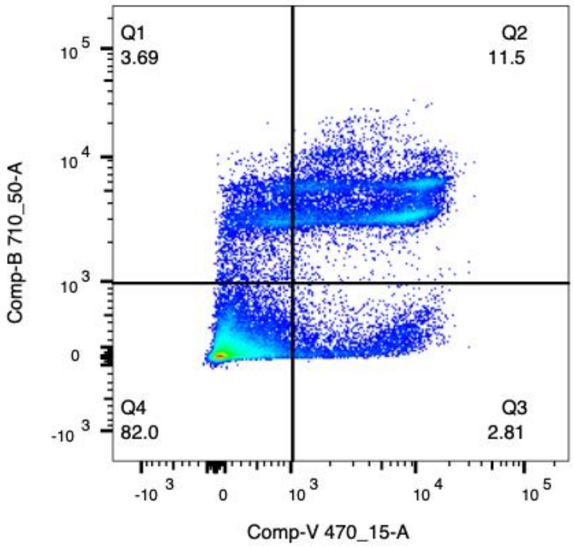

50 µM Benomyl

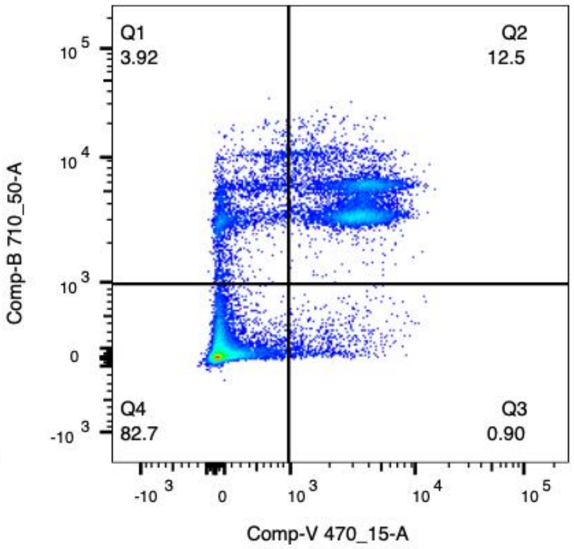

50 µM Dexamethasone

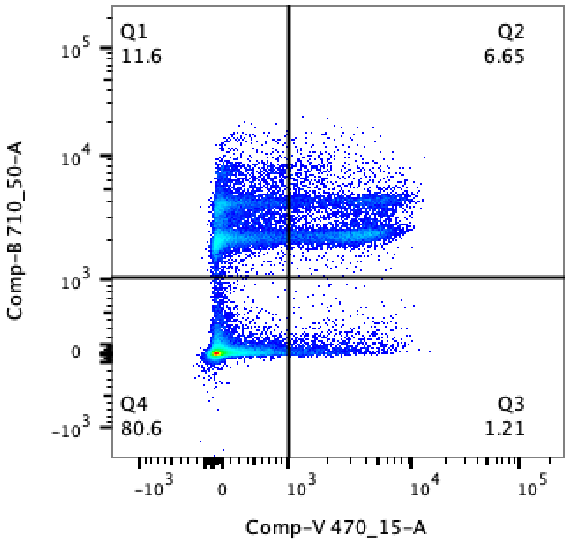

1 mM Dopamine

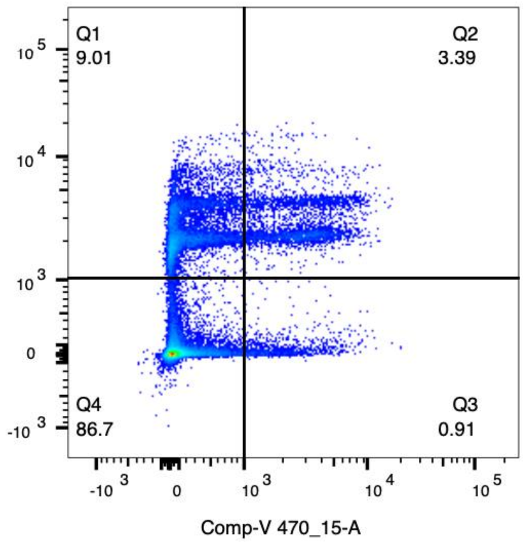

1 mM Norepinephrine

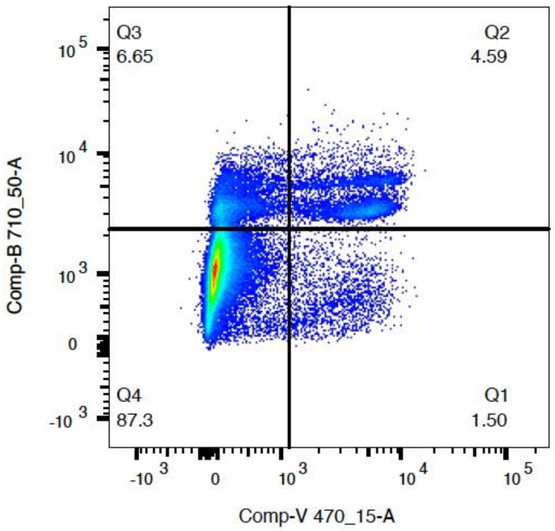

10 μM **3a**

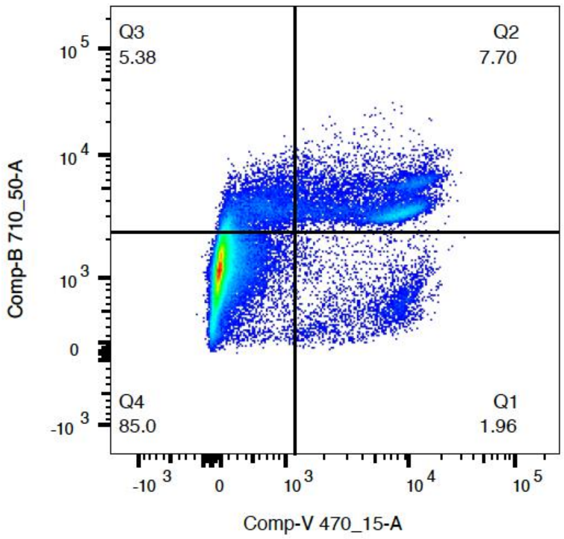

20 μM **3a**

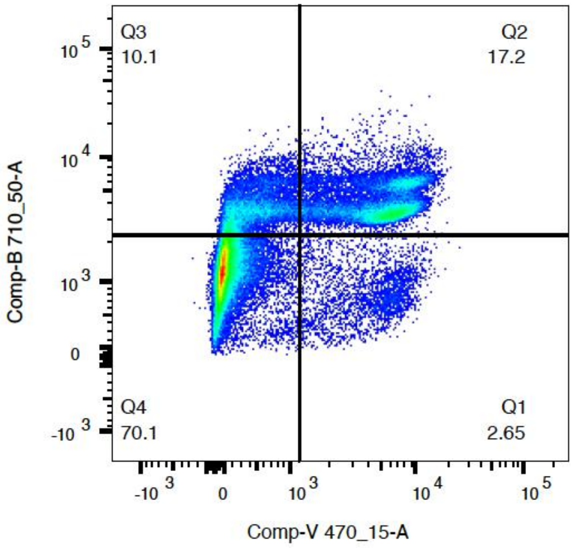

30 μM **3a**

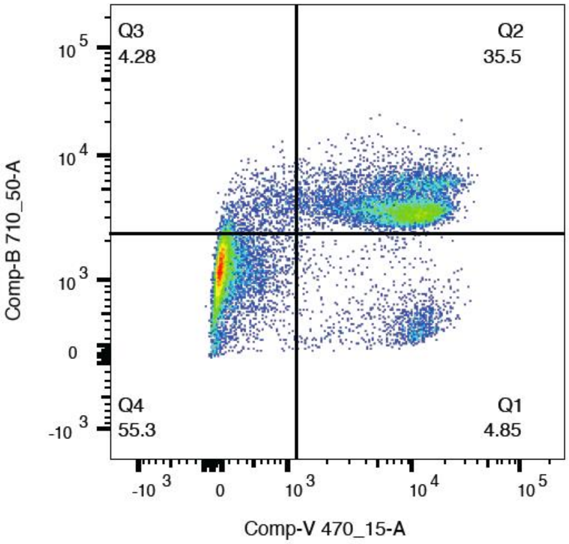

40 μM **3a**

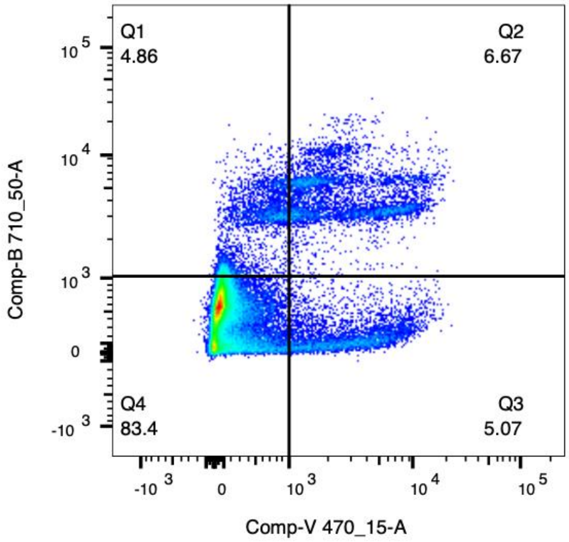

10 μM **3b**

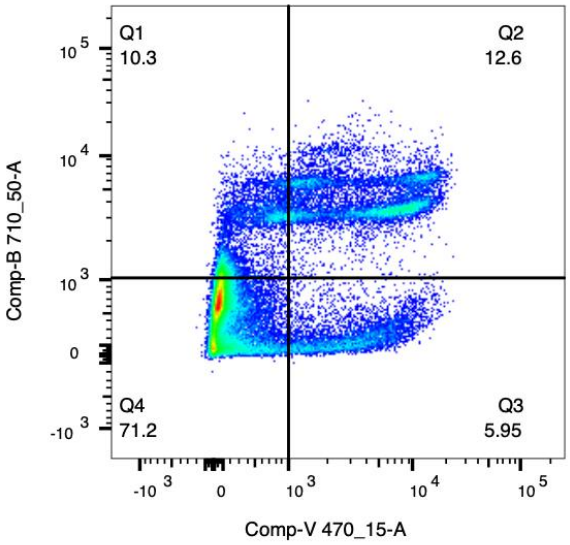

20 μM **3b**

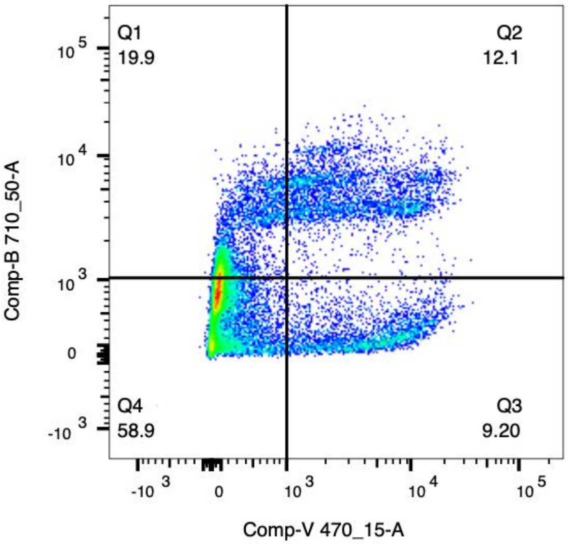

30 µM **3b**

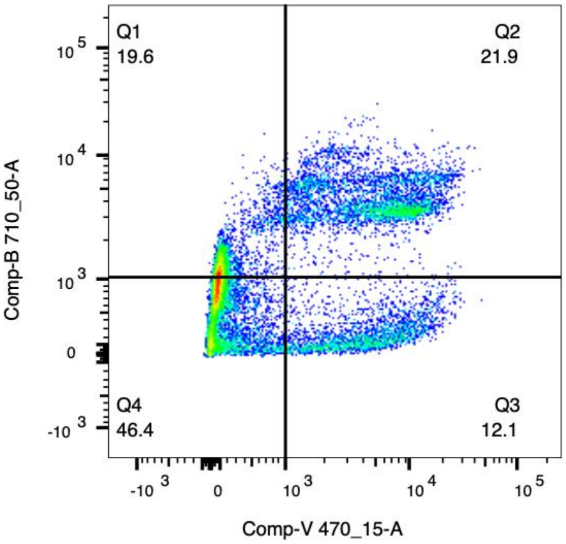

40 µM **3b**

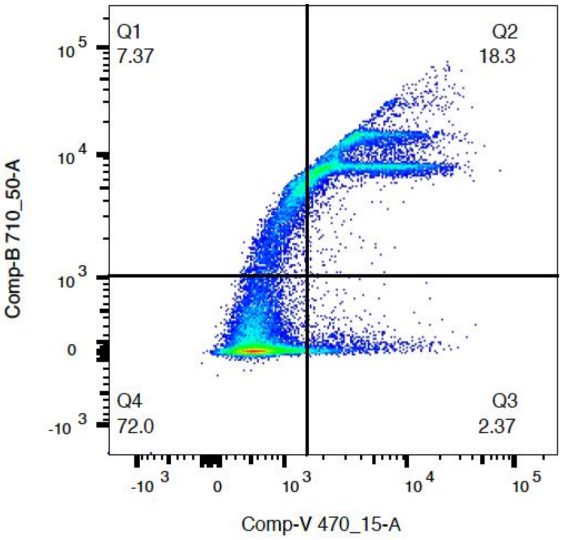

10 µM **1a,** 20 µM **3a**, 50 µM Ben, 50 µM Dexa, 1 mM Dopamine

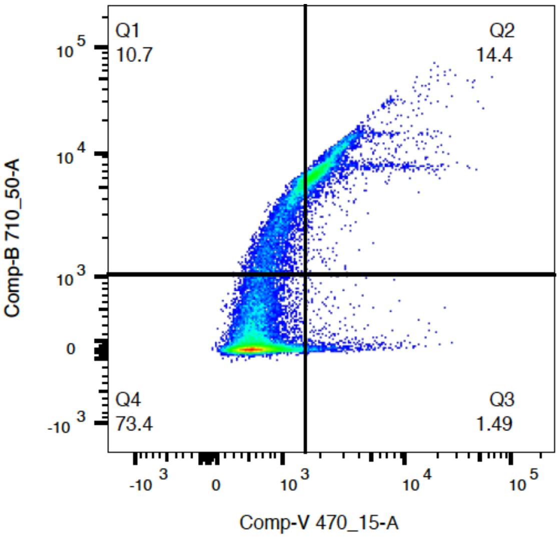

10 µM **1a,** 20 µM **3a**, 50 µM Ben, 50 µM Dexa, 1 mM Norepinephrine

XII: Supplementary Figure 7: Localization of Probe 1a and 3a in U-87 MG cells

Cells were plated in an IBIDI 8-well glass bottom chamber at a density of 25,000 cells per well in media and allowed to adhere overnight at 37 °C, 5% CO_2. Fresh 10 mM stock solutions of probes **1a** and **3a** were prepared in DMSO on the day of experimentation. Working solutions of all compounds in media were prepared the day of experimentation. Media was removed from wells and 200 μL of 10 μM **1a** was added to desired wells and placed in an incubator for 1.5 hours. Media was removed from wells and 200 μL of 20 μM **3a** was added to desired wells and placed in an incubator for 15 minutes. Then, probe media was removed, and cells were washed with 200 μL of PBS for 5 minutes in the incubator (repeated 3x). Cells were then stained with either LysoTracker® RED DND-99 (ThermoFischer, L7528) or Mitrotracker® FM (ThermoFischer, M22425) was added according to manufacturer protocol, and cells were incubated for 20 minutes before staining media was removed and cells were washed with 200 μL of PBS for 5 minutes (repeated 3 times). PBS was removed and replaced with 200 μL of fresh media followed by immediate imaging. Five images were captured for each well. Colocalization analysis for Pearsons' R (R) and Mander's Colocalization Coefficient (MCC) were conducted using the EzColocalization Plugin for ImageJ.[6] The scale bar represents 10 μm. The layout for this experiment is depicted below where x represents a blank well:

**1a** + MitoTracker	**3a** + MitoTracker	**X**	**X**
**1a** + LysoTracker	**3a** + LysoTracker	**X**	**X**

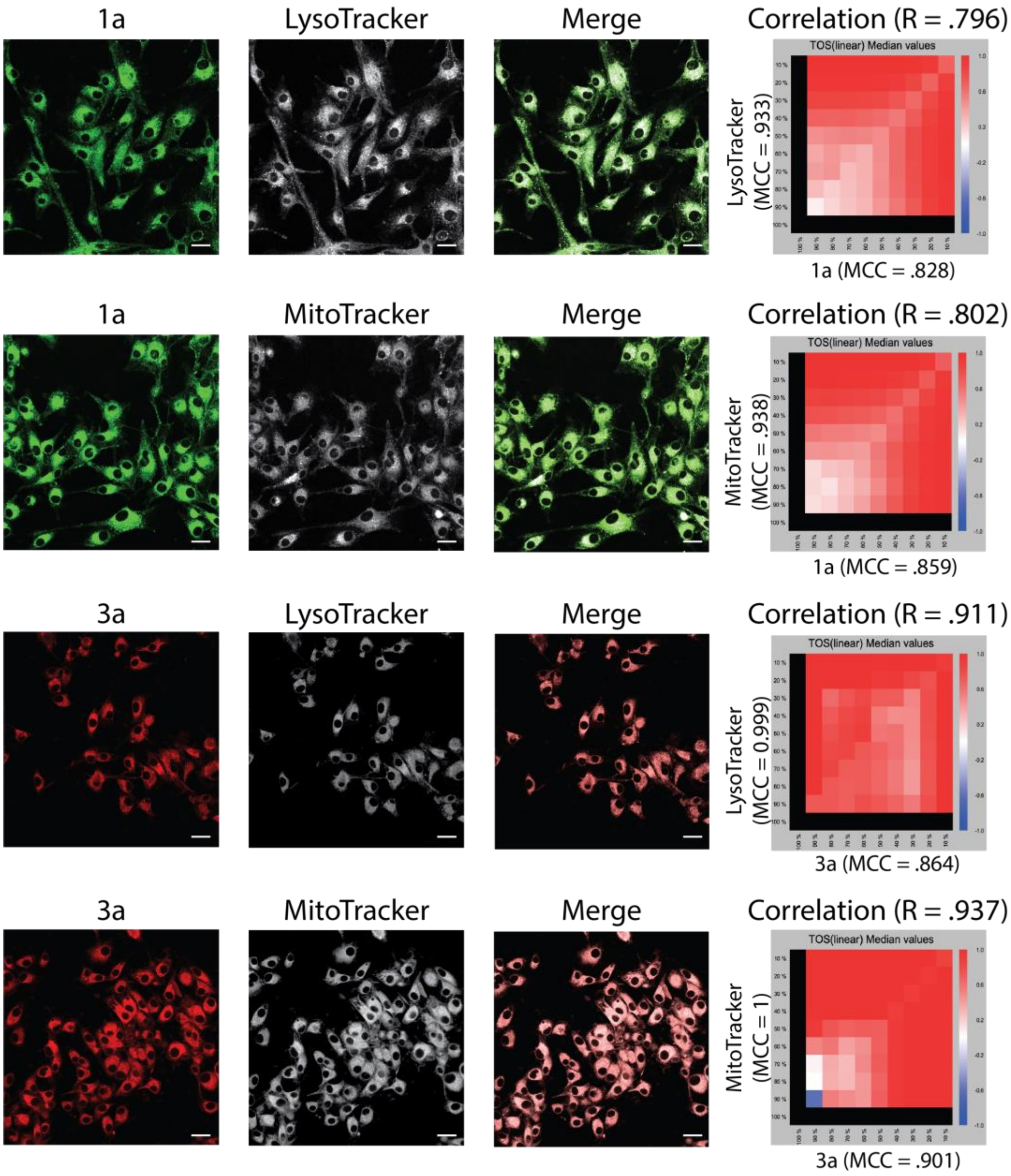

XIII: Supplementary Figure 8: FLIM-FRET Imaging of U-87 MG cells with biological controls

Cells were plated in an IBIDI 8-well glass bottom chamber at a density of 25,000 cells per well in media and allowed to adhere overnight at 37 °C, 5% CO_2. Fresh 10 mM stock solutions of probes **1a**, **2a**, and **3a** were prepared in DMSO on the day of experimentation. Working solutions of all compounds in PBS were prepared the day of experimentation to final concentrations of 20 mM glucose, 1 mM catechol, and 1% v/v H_2O_2. Media was removed from wells and washed with 200 μL of PBS for 5 minutes in the incubator (repeated 3x total). PBS was removed from wells and 200 μL of the glucose or catechol

was added to desired wells. Cells were placed in an incubator for 15 minutes, followed by removal of the dosage PBS. Solutions of probe **1a** in combination with catechol or glucose or **2a** alone were added to desired cells and placed in an incubator for 2 hours. Next, dosage PBS was removed, and cells were washed with 200 μL of PBS for 5 minutes in the incubator. PBS was removed, and 200 μL of the 20 μM solution of either probe **3a** in PBS or PBS was added and placed in the incubator for 15 minutes, followed by removal of the dosage PBS and washing with 200 μL of PBS for 5 minutes in the incubator. PBS was removed and replaced with 200 μL of fresh PBS followed by immediate imaging. 5 images were captured for each well. This process was repeated in triplicate on separate days with different cell passage numbers. Statistical analysis was conducted via Student's T-test (n = 15). An outlier test was performed to remove any extraneous data points. Error bars represent standard deviation. The layout for this experiment is depicted below where x represents a blank well:

**2a**	**1a** + **3a**	**1a** + **3a**<br>+ glucose	**1a** + **3a**<br>+ catechol
**1a** + **3a**<br>+ H_2O_2	**X**	**X**	**X**

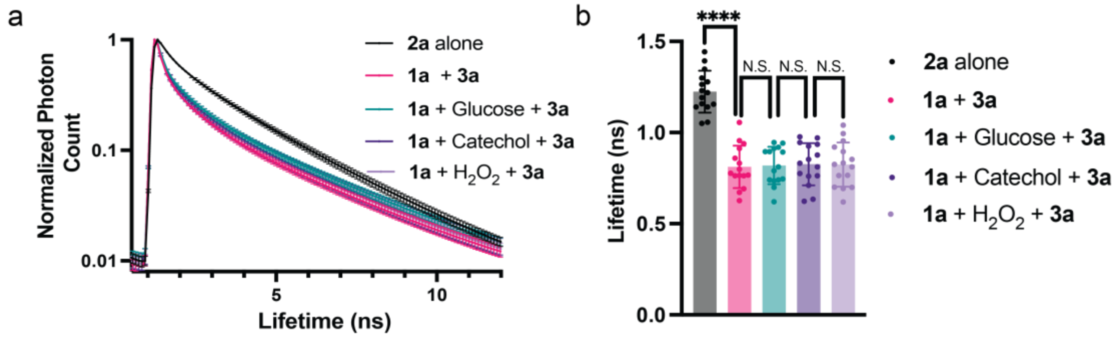

XIV: Supplementary Figure 9: FLIM-FRET Imaging of U-87 MG cells with FRET donor 2a and negative controls 2b, 2c, and 2d with FRET acceptor 3a

Cells were plated in an IBIDI 8-well glass bottom chamber at a density of 25,000 cells per well in media and allowed to adhere overnight at 37 °C, 5% CO_2. Fresh 10 mM stock solutions of probes **2a**, **2b**, **2c**, **2d**, and **3a** were prepared on the day of experimentation. 10 μM solutions of probes **2a**, **2b**, **2c**, and **2d** were prepared by diluting 0.8 μL of 10 mM stock in 799.2 μL of culturing media. Probe **3a** was prepared similarly except to a 20 μM concentration (1.6 μL of 10 mM stock in 998.4 μL of culturing media). Media was removed from wells, and 200 μL of the 10 μM solutions of either probe **2a**, **2b**, **2c**, or **2d** in media was added to the wells. Cells were placed in an incubator for 2 hours, followed by removal of the dosage media and washed with 200 μL of PBS for 5 minutes in the incubator. PBS was removed, and 200 μL of the 20 μM solution of either probe **3a** or media was added and placed in the incubator for 15 minutes, followed by removal of the dosage media and washing with 200 μL of PBS for 5 minutes in the incubator. PBS was removed and replaced with 200 μL of standard culture media followed by immediate microscopy. 5 images were captured for each well. This process was repeated in triplicate on separate days with

different cell passage numbers. Statistical analysis was conducted via Student's T-test (n = 15). An outlier test was performed to remove any extraneous data points. Error bars represent standard deviation. The layout for this experiment is depicted below where x represents a blank well:

**2a**	**2b**	**2c**	**2d**
**2a + 3a**	**2b + 3a**	**2c + 3a**	**2d + 3a**

Graph of lifetimes mirroring Figure 4b-c can be seen below.

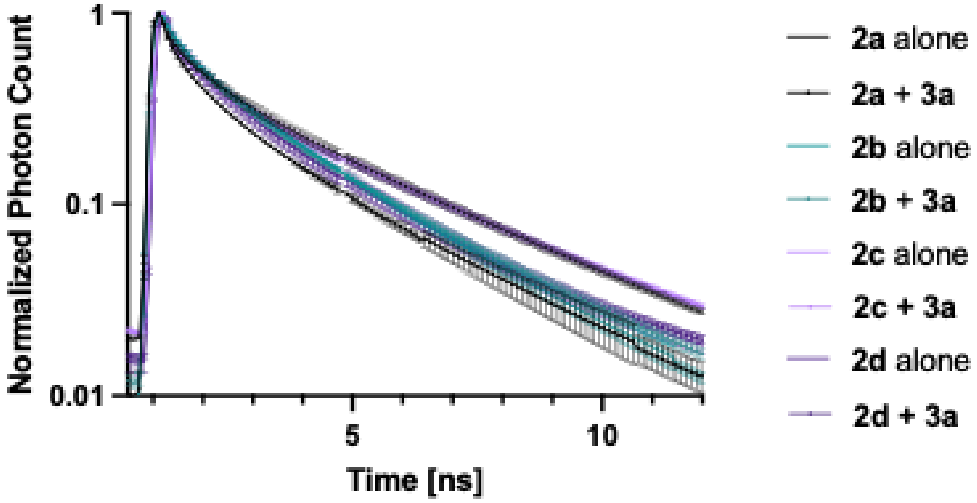

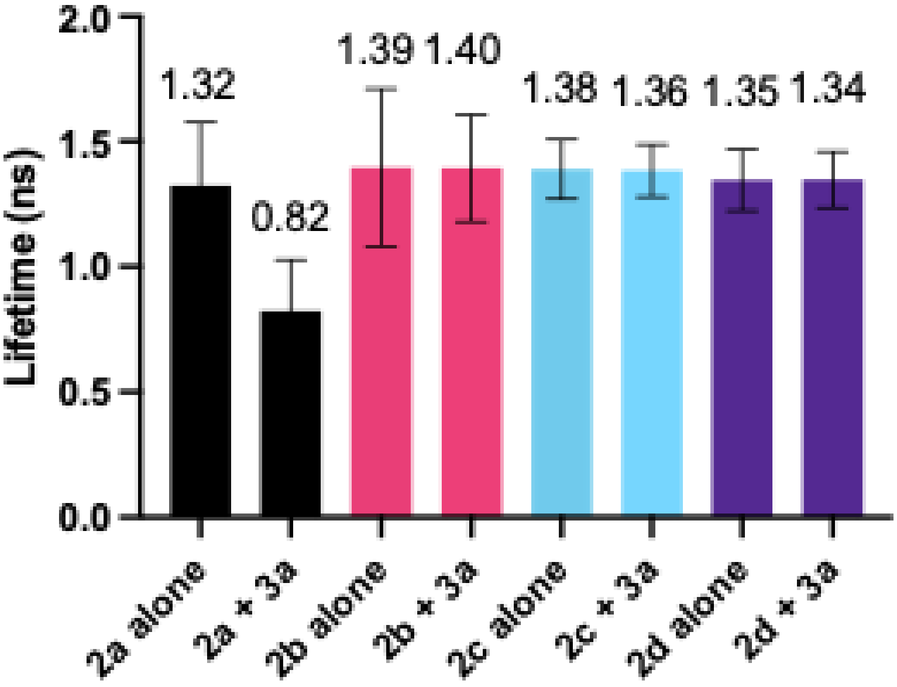

XV: Supplementary Figure 10: FLIM-FRET Imaging of U-87 MG cells with exogenous dopamine

Cells were plated in an IBIDI 8-well glass bottom chamber at a density of 25,000 cells per well in media and allowed to adhere overnight at 37 °C, 5% CO_2. Fresh 10 mM stock solutions of probes **1a**, **2a**, and **3a** were prepared in DMSO on the day of experimentation. All activators and inhibitor stocks were prepared fresh weekly: 50 mM dopamine (DA), 10 mM DDZ, 10 mM Dexa, and 10 mM Ben. Working solutions of all compounds in media were prepared the day of experimentation to final concentrations of 1 mM DA, 50 μM DDZ, 25 μM Dexa, and 50 μM Ben. Media was removed from wells, and 200 μL of the drugs was added to desired wells. Cells were placed in an incubator for 15 minutes, followed by removal of the dosage media. Solutions of probe **1a** in combination with drugs or **2a** alone were added to desired cells and placed in an incubator for 2 hours. Next, dosage media was removed, and cells were washed with 200 μL of PBS for 5 minutes in the incubator. PBS was removed, and 200 μL of the 20 μM solution of either probe **3a** or media was added and placed in the incubator for 15 minutes, followed by removal of the dosage media and washing with 200 μL of PBS for 5 minutes in the incubator. PBS was removed and replaced with 200 μL of standard culture media followed by immediate imaging. 5 images were captured for each well. This process was repeated in triplicate on separate days with different cell passage numbers. Statistical analysis was conducted via Student's T-test (n = 15). An outlier test was performed to remove any extraneous data points. Error bars represent standard deviation. The layout for this experiment is depicted below where x represents a blank well:

**2a**	**1a** + DA + **3a**	**1a** + DA + DDZ + **3a**	**1a** + DA + Dexa + **3a**
**1a** + DA + Ben + **3a**	**1a** + DA + DDZ + Ben + Dexa + **3a**	**X**	**X**

Graph of lifetimes mirroring Figure 5a-b can be seen below with extended axes and additional labels.

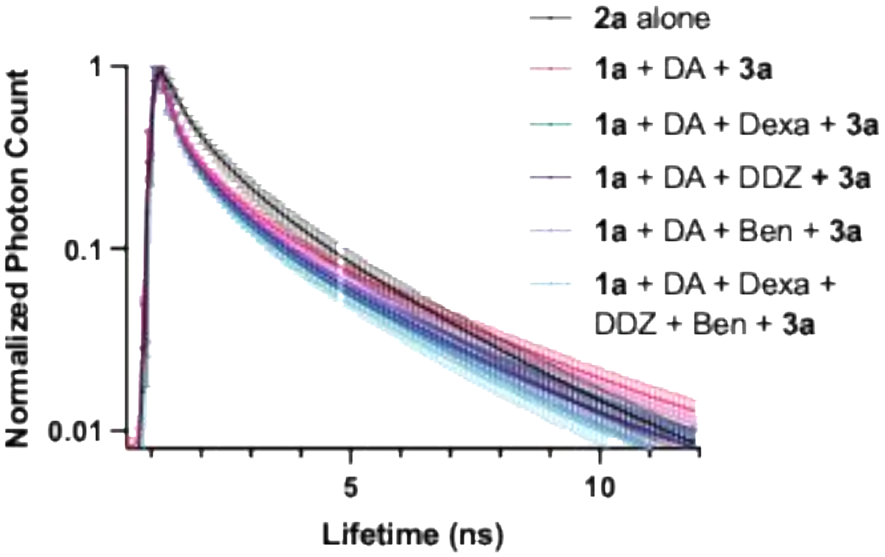

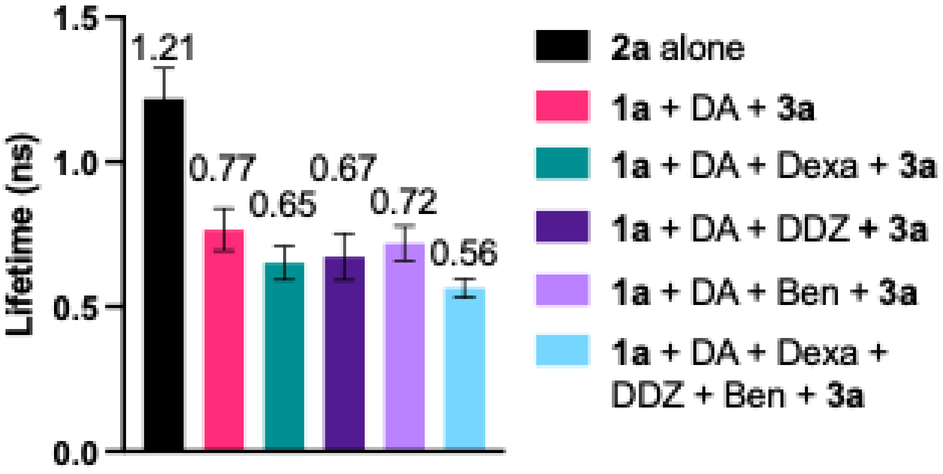

XVI: Supplementary Figure 11: FLIM-FRET Imaging of U-87 MG cells with exogenous norepinephrine

Cells were plated in an IBIDI 8-well glass bottom chamber at a density of 25,000 cells per well in media and allowed to adhere overnight at 37 °C, 5% CO_2. Fresh 10 mM stock solutions of probes **1a**, **2a**, and **3a** were prepared in DMSO on the day of experimentation. All activators and inhibitor stocks were prepared fresh weekly: 50 mM norepinephrine (NE), 10 mM DDZ, 10 mM Dexa, and 10 mM Ben. Working solutions of all compounds in media were prepared the day of experimentation to final concentrations of 1 mM NE, 50 µM DDZ, 25 µM Dexa, and 50 µM Ben. Media was removed from wells, and 200 µL of the drugs was added to desired wells. Cells were placed in an incubator for 15 minutes, followed by removal of the dosage media. Solutions of probe **1a** in combination with drugs or **2a** alone were added to desired cells and placed in an incubator for 2 hours. Next, dosage media was removed, and cells were washed with 200 µL of PBS for 5 minutes in the incubator. PBS was removed, and 200 µL of the 20 µM solution of either probe **3a** or media was added and placed in the incubator for 15 minutes, followed by removal of the dosage media and washing with 200 µL of PBS for 5 minutes in the incubator. PBS was removed and replaced with 200 µL of standard culture media followed by immediate imaging. 5 images were captured for each well. This process was repeated in triplicate on separate days with different cell passage numbers. Statistical analysis was conducted via Student's T-test (n = 15). An outlier test was performed to remove any extraneous data points. Error bars represent standard deviation. The layout for this experiment is depicted below where x represents a blank well:

**2a**	**1a** + **NE** + **3a**	**1a** + NE + DDZ + **3a**	**1a** + NE + Dexa + **3a**
**1a** + NE + Ben + **3a**	**1a** + NE + DDZ + Ben + Dexa + **3a**	**X**	**X**

Graph of lifetimes mirroring Figure 5d-e can be seen below with extended axes and additional labels.

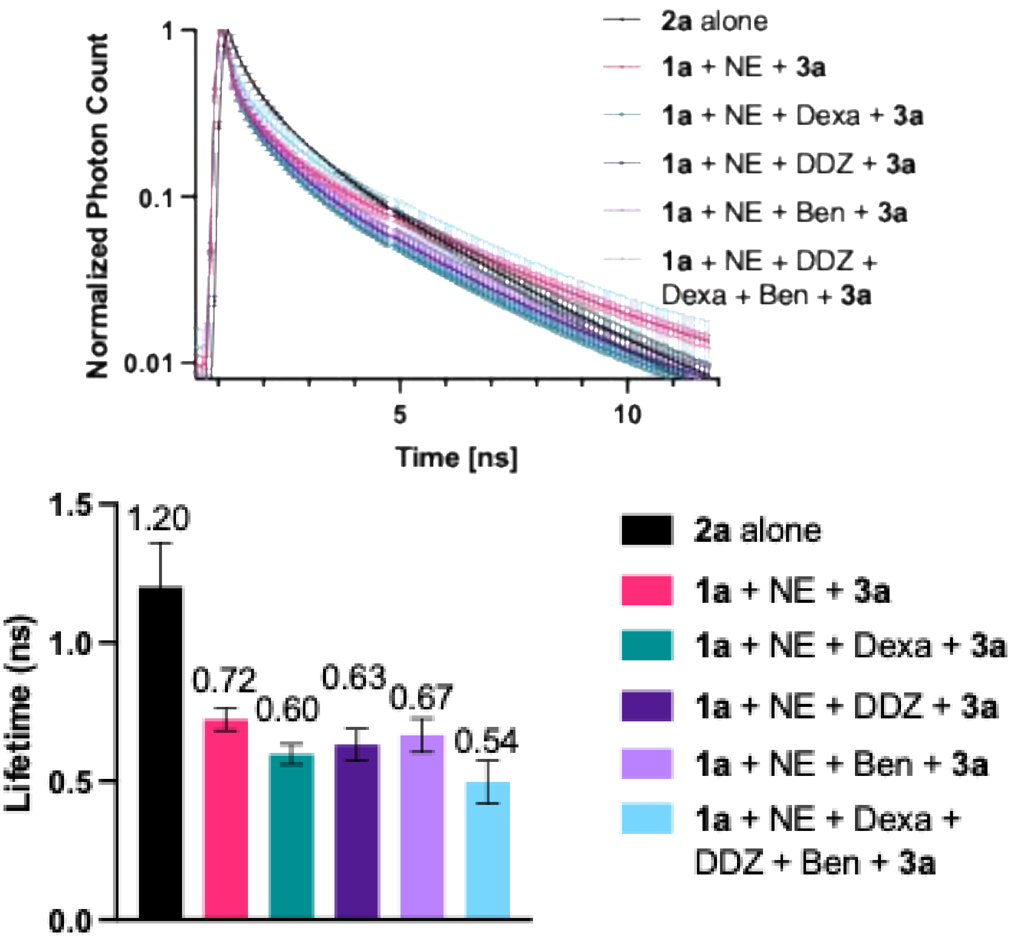

XVII: Supplementary Figure 12: FLIM-FRET Imaging of U-87 MG cells endogenous DOPAL/DOPEGAL

Cells were plated in an IBIDI 8-well glass bottom chamber at a density of 25,000 cells per well in media and allowed to adhere overnight at 37 °C, 5% CO_2. Fresh 10 mM stock solutions of probes **1a**, **2a**, and **3a** were prepared in DMSO on the day of experimentation. All activators and inhibitor stocks were prepared fresh weekly: 10 mM DDZ, 10 mM Dexa, and 10 mM Ben. Working solutions of all compounds in media were prepared the day of experimentation to final concentrations of 50 µM DDZ, 25 µM Dexa, and 50 µM Ben. Media was removed from wells, and 200 µL of working solutions of probe **1a** in combination with drugs or **2a** alone were added to desired cells and placed in an incubator for 2 hours. Next, dosage media was removed, and cells were washed with 200 µL of PBS for 5 minutes in the incubator. PBS was removed, and 200 µL of the 20 µM solution of either probe **3a** or media was added and placed in the incubator for 15 minutes, followed by removal of the dosage media and washing with 200 µL of PBS for 5 minutes in the incubator. PBS was removed and replaced with 200 µL of standard culture media followed by immediate imaging. 5 images were captured for each well. This process was repeated in triplicate on separate days with different cell passage numbers. Statistical analysis was

conducted via Student's T-test (n = 15). An outlier test was performed to remove any extraneous data points. Error bars represent standard deviation. The layout for this experiment is depicted below where x represents a blank well:

**2a**	**1a** + **3a**	**1a** + DDZ + **3a**	**1a** + Dexa + **3a**
**1a** + Ben + **3a**	**1a** + DDZ + Ben + Dexa + **3a**	**X**	**X**

Graph of lifetimes mirroring Figure 6a-b can be seen below with extended axes and additional labels.

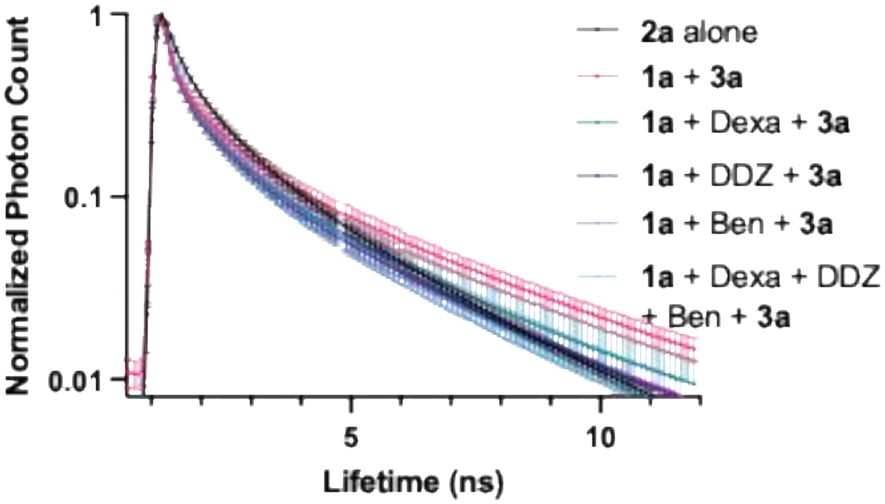

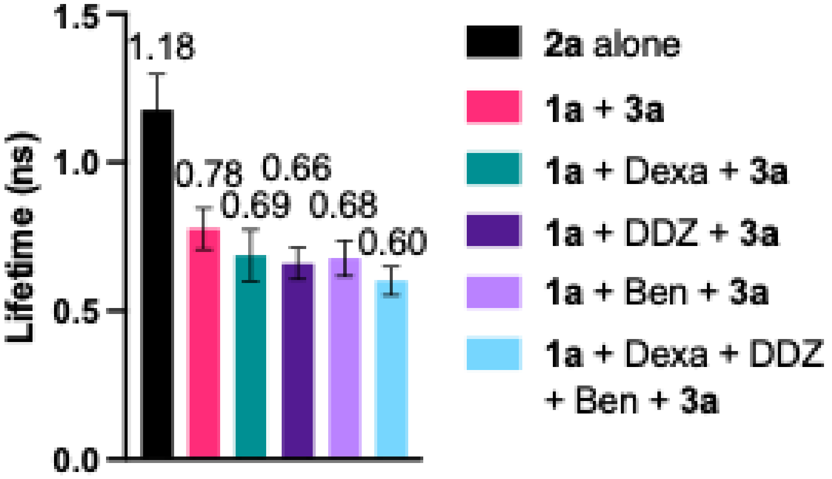

XVIII: Supplementary Figure 13: FLIM-FRET Imaging of DBH heterozygote and KO mice

DBH +/- and DBH -/- mice were anesthetized with isoflurane and euthanized by rapid decapitation. Mouse brains were rapidly dissected on ice and flash-frozen in isopentane on dry ice. Samples were stored at -20 °C for 24 hours. Brains were transferred to 5 mL centrifuge tubes containing 10 μM probe **1a** in PBS (1.5 μL of 10 mM stock of probe **1a** in DMSO into 1498.5 μL of PBS) and allowed to diffuse for 16 hours in a 4 °C refrigerator. Solution was removed and replaced with 20 μM probe **3a** in PBS (3 μL of 10 mM stock of probe **3a** in DMSO into 1497 μL of PBS) and allowed to diffuse for 8 hours in a 4 °C refrigerator. Solution was removed and brains were fixed with paraformaldehyde. Brains were embedded in OT medium (Tissue-Tek) and sectioned by cryostat into 60 µM thick coronal sections at the level of the LC. Sections were immediately transferred to glass Superfrost Plus slides which were then cover slipped with Fluoromount-G (Southern Biotech, Birmingham, AL) and allowed to dry before imaging.

XIX: References

[1] Thomas, S. A.; Marck, B. T.; Palmiter, R. D.; Matsumoto, A. M. Restoration of norepinephrine and reversal of phenotypes in mice lacking dopamie beta-hydroxylase. *J. Neurochem.* **1998**, *70,* 1471.

[2] Thomas, S. A.; Matsumoto, A. M.; Palmiter, R. D. Noradrenaline is essential for mouse fetal development. *Nature* **1995**, *374,* 643-646.

[3] Bourdélat-Parks, B. N.; Anderson, G. M.; Donaldson, Z. R.; Weiss, J. M.; Bonsall, R. W.; Emery, M. S.; Liles, L. C.; Weinshenker, D. Effects of dopamine beta-hydroxylase genotype disulfiram inhibition on catechol homeostatis in mice. *Physchopharmacology (Berl.)* **2005**, *183*, 72-80.

[4] Szot, P.; Weinshenker, D.; White, S. S.; Robbins, C. A.; Rust, N. C.; Schwartzkroin, P. A.; Palmiter, R. D. Norepinephrine-deficient mice have increased susceptibility to seizure-inducing stimuli. *J. Neurosci*. **1999**, *19*, 10985-10992.

[5] Kaithal, A.; Wagener, T.; Bellotti, P.; Daniliuc, G.; Schlichter, L.; Glorius. F. *Angew. Chem., Int. Ed.* **2022**, *32*, e202206687.

[6] Stauffer, W.; Sheng, H.; Lin, H. N. EzColocalization: An ImageJ plugin for visualizing and measuring colocalization in cells and organisms. *Sci. Rep.* **2018**, *8*, 15764.

XX: ^{1}H and ^{13}C NMR spectra of synthesized compounds.

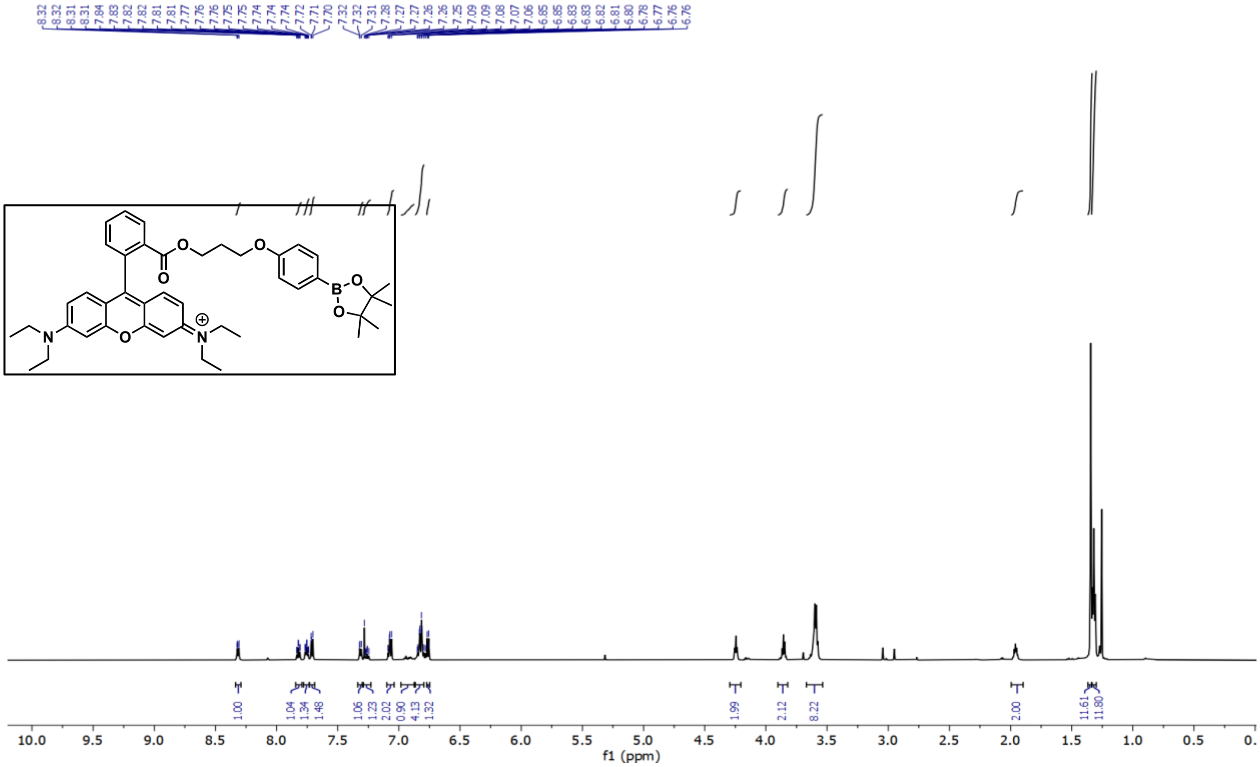

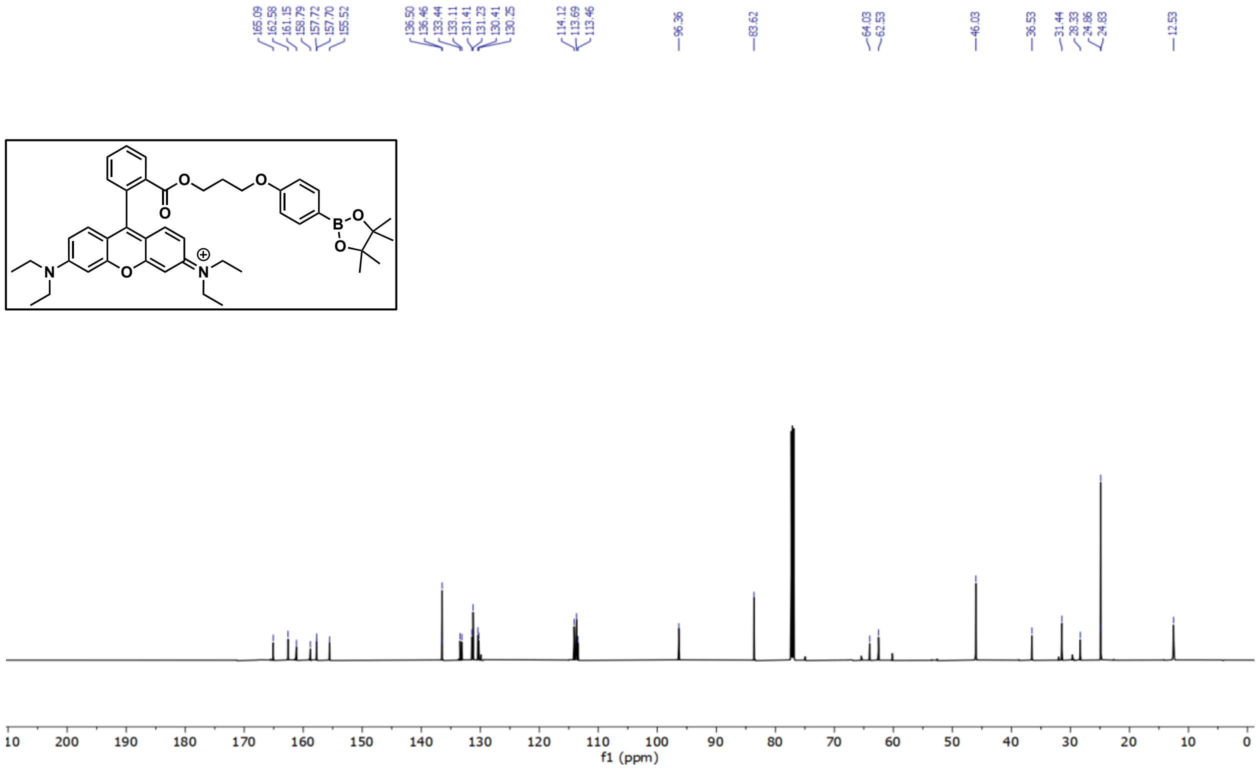

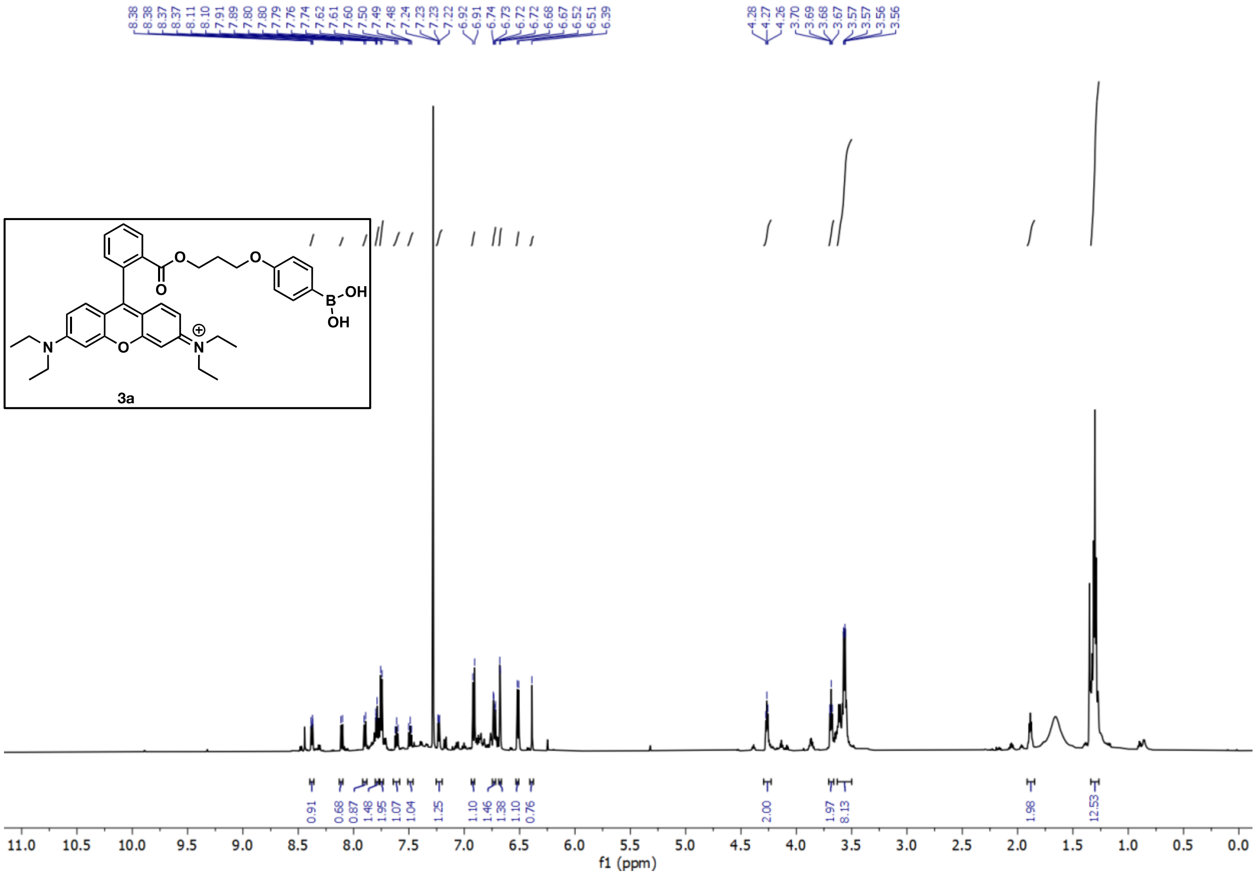

3a
f1 (ppm)

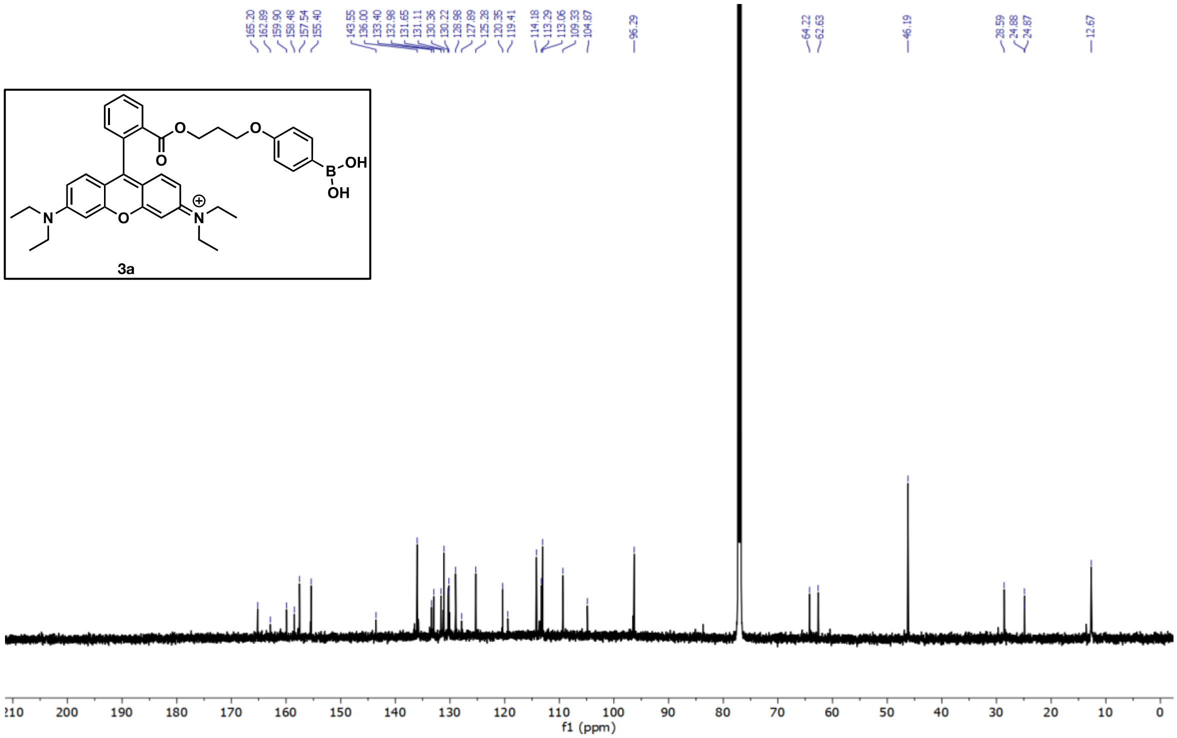

3a
f1 (ppm)

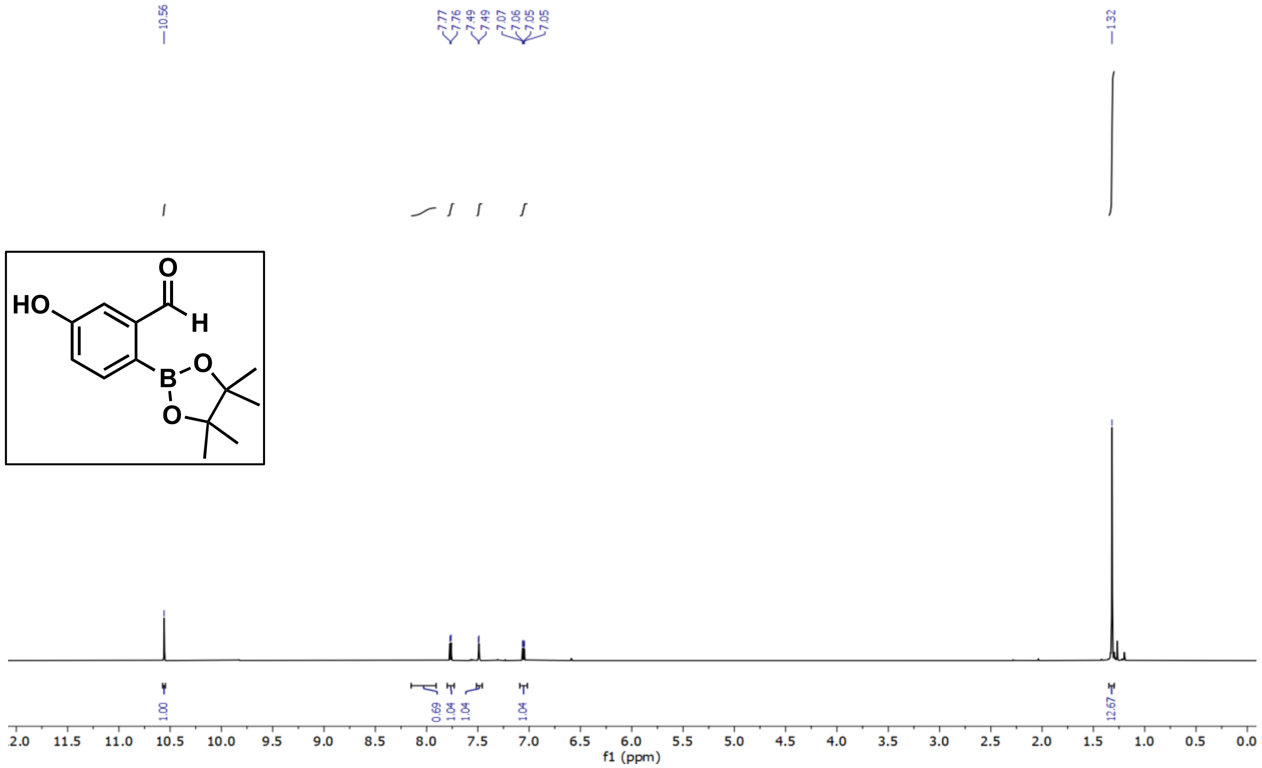
10.56
7.77
7.76
7.49
7.49
7.07
7.06
7.05
7.05
1.32
HO
O
H
B
O
O
1.00
0.69
1.04
1.04
1.04
12.67
f1 (ppm)

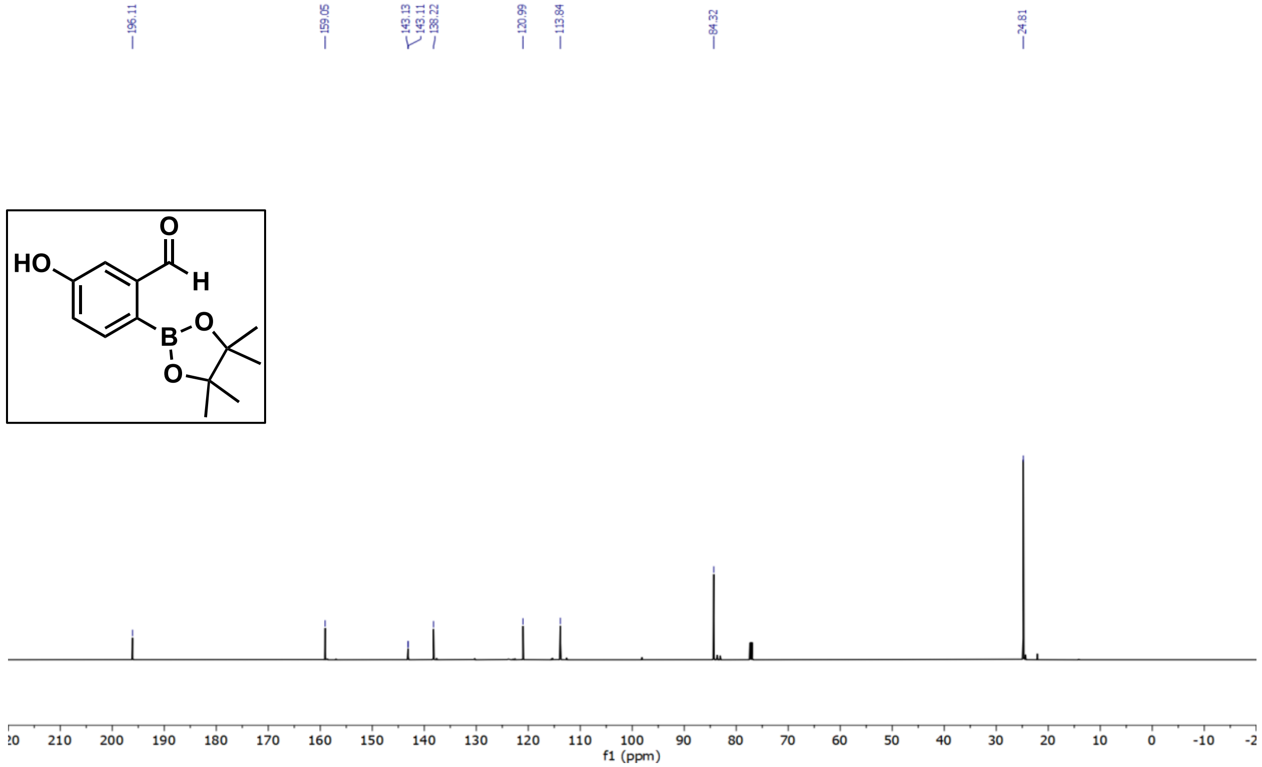
196.11
159.05
143.13
143.11
138.22
120.99
113.84
84.32
24.81
HO
O
H
B
O
O
f1 (ppm)

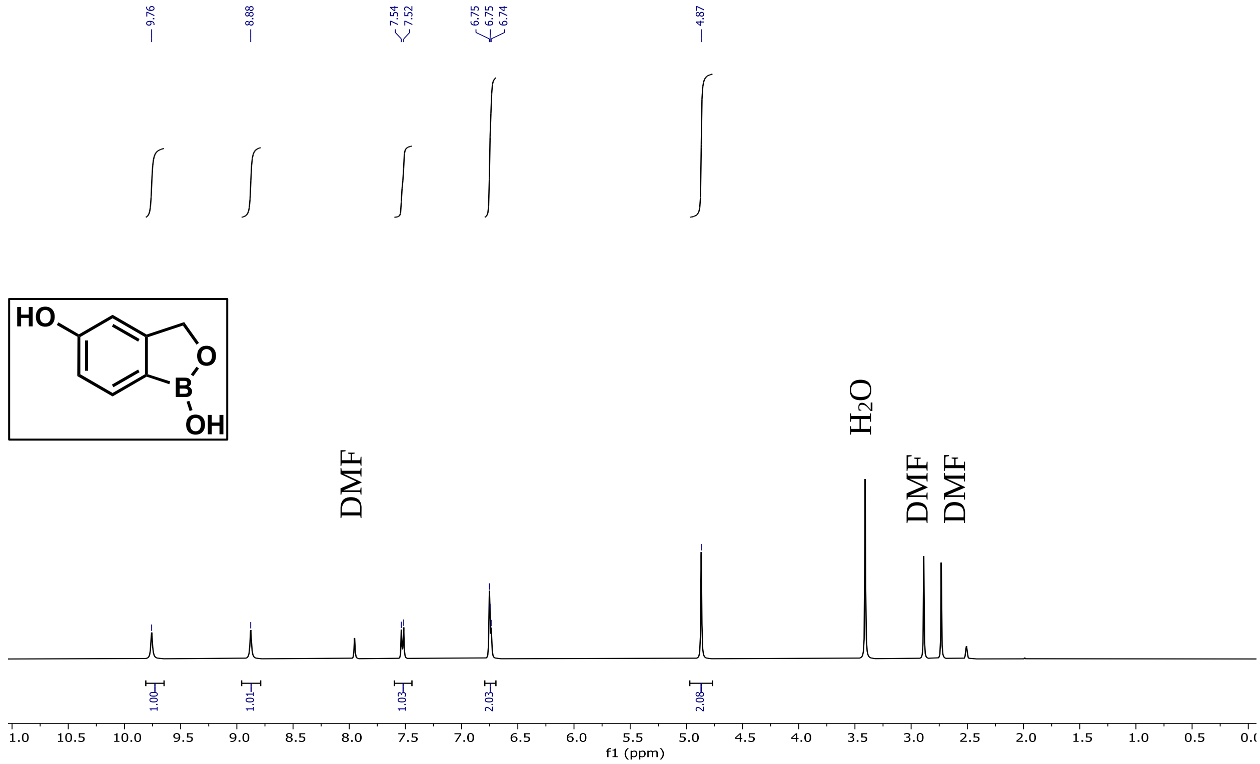

9.76
8.88
7.54
7.52
6.75
6.75
6.74
4.87
HO
O
B
OH
DMF
H2O
DMF
DMF
1.00
1.01
1.03
2.03
2.08
f1 (ppm)

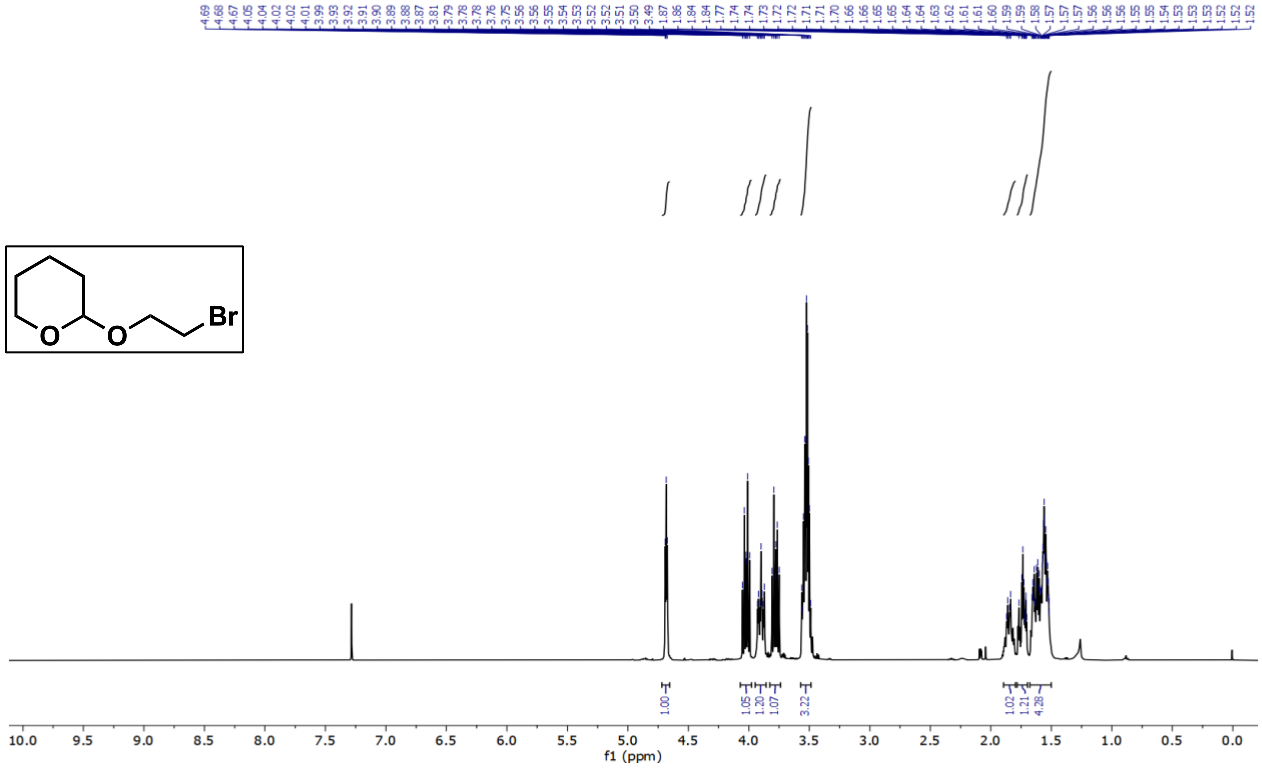
O
O
Br
1.00
1.05
1.20
1.07
3.22
1.02
1.21
4.28
f1 (ppm)

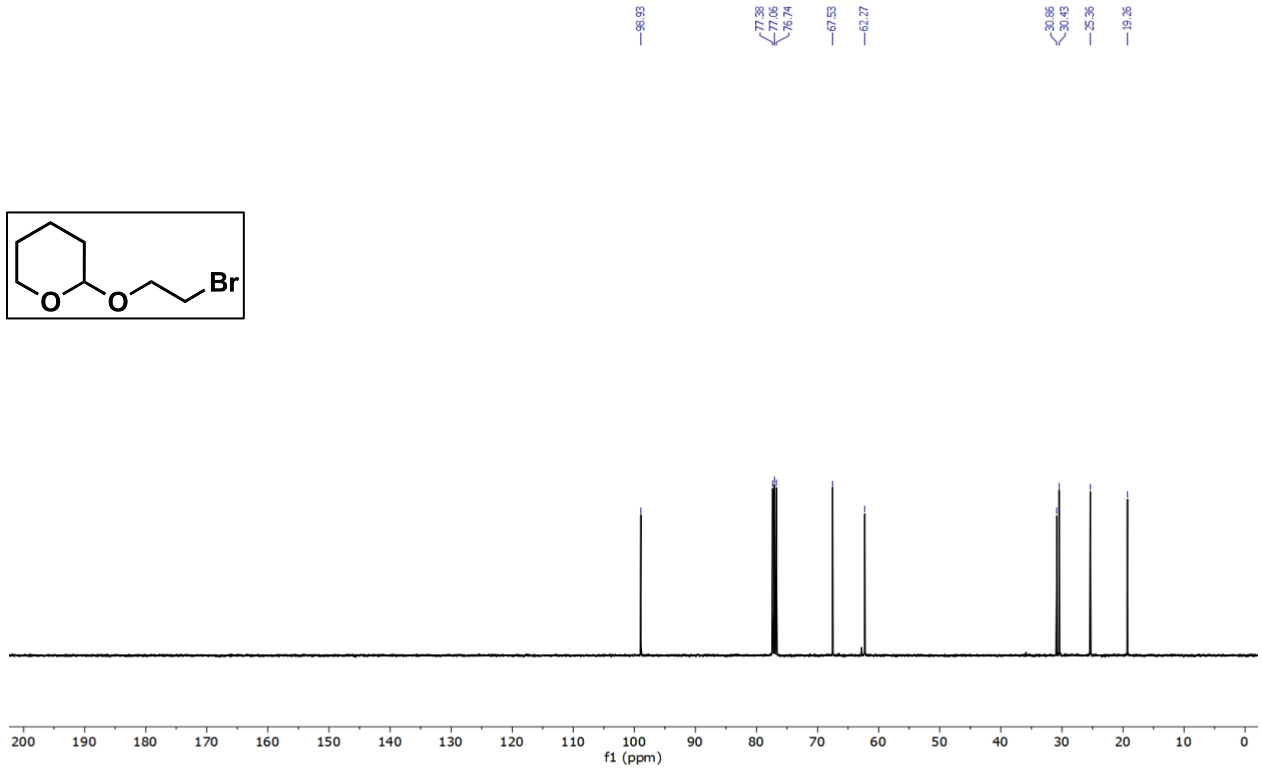
98.93
77.38
77.06
76.74
67.53
62.27
30.86
30.43
25.36
19.26
O
O
Br
f1 (ppm)

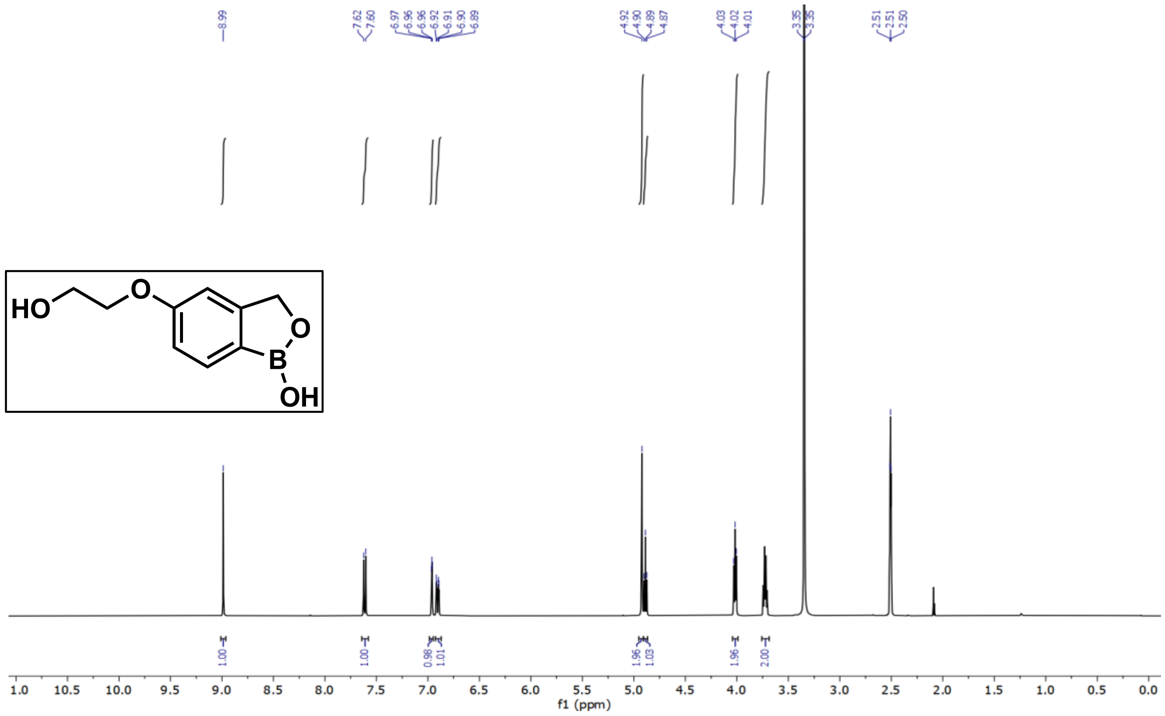
8.99
7.62
7.60
6.97
6.96
6.96
6.92
6.91
6.90
6.89
4.92
4.90
4.89
4.87
4.03
4.02
4.01
3.35
3.35
2.51
2.51
2.50
HO
O
O
B
OH
1.00
1.00
0.98
1.01
1.96
1.03
1.96
2.00
1.0 10.5 10.0 9.5 9.0 8.5 8.0 7.5 7.0 6.5 6.0 5.5 5.0 4.5 4.0 3.5 3.0 2.5 2.0 1.5 1.0 0.5 0.0
f1 (ppm)

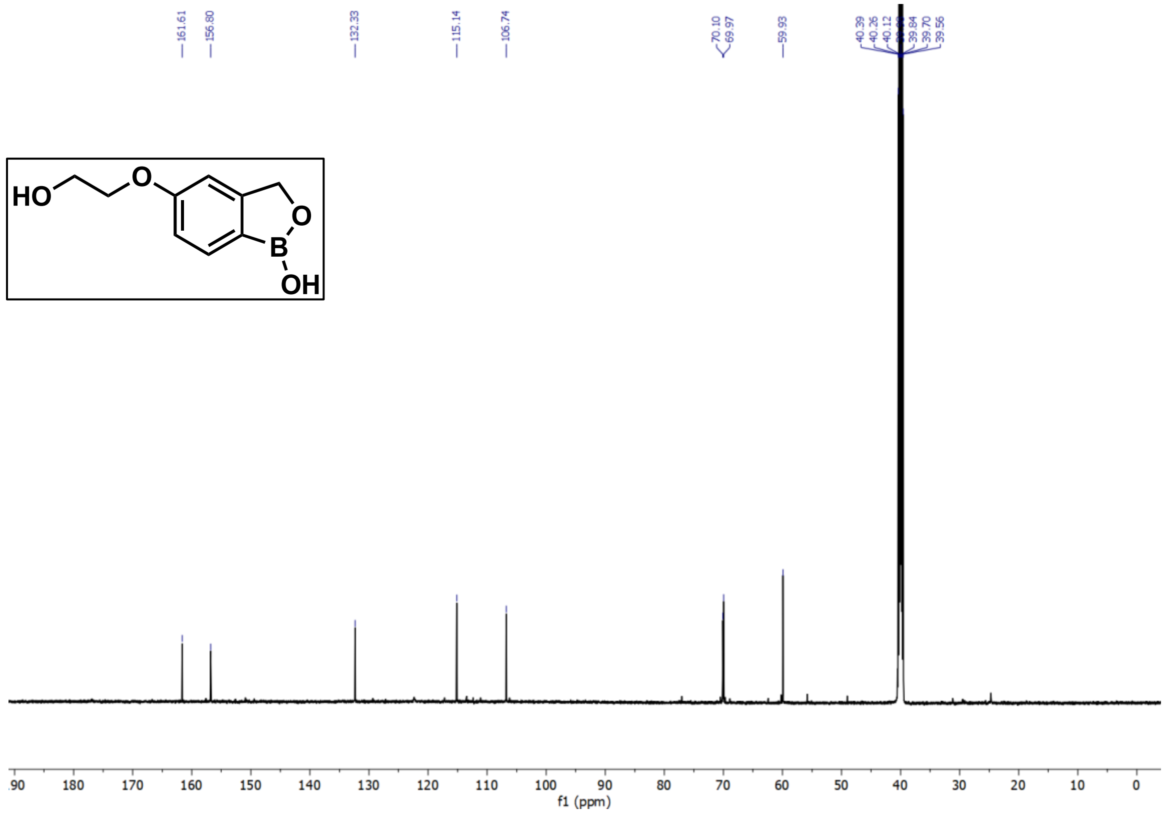
161.61
156.80
132.33
115.14
106.74
70.10
69.97
59.93
40.39
40.26
40.12
39.84
39.70
39.56
HO
O
O
B
OH
90 180 170 160 150 140 130 120 110 100 90 80 70 60 50 40 30 20 10 0
f1 (ppm)

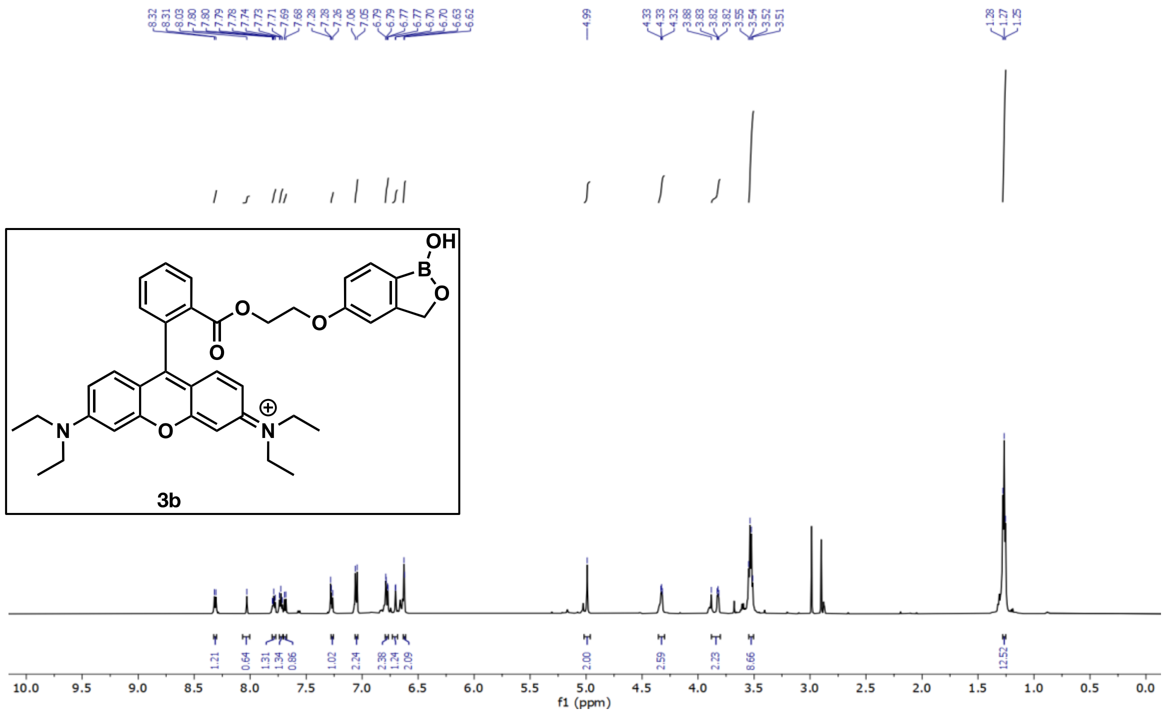
OH
B
O
O
O
O
N
O
N
3b
f1 (ppm)

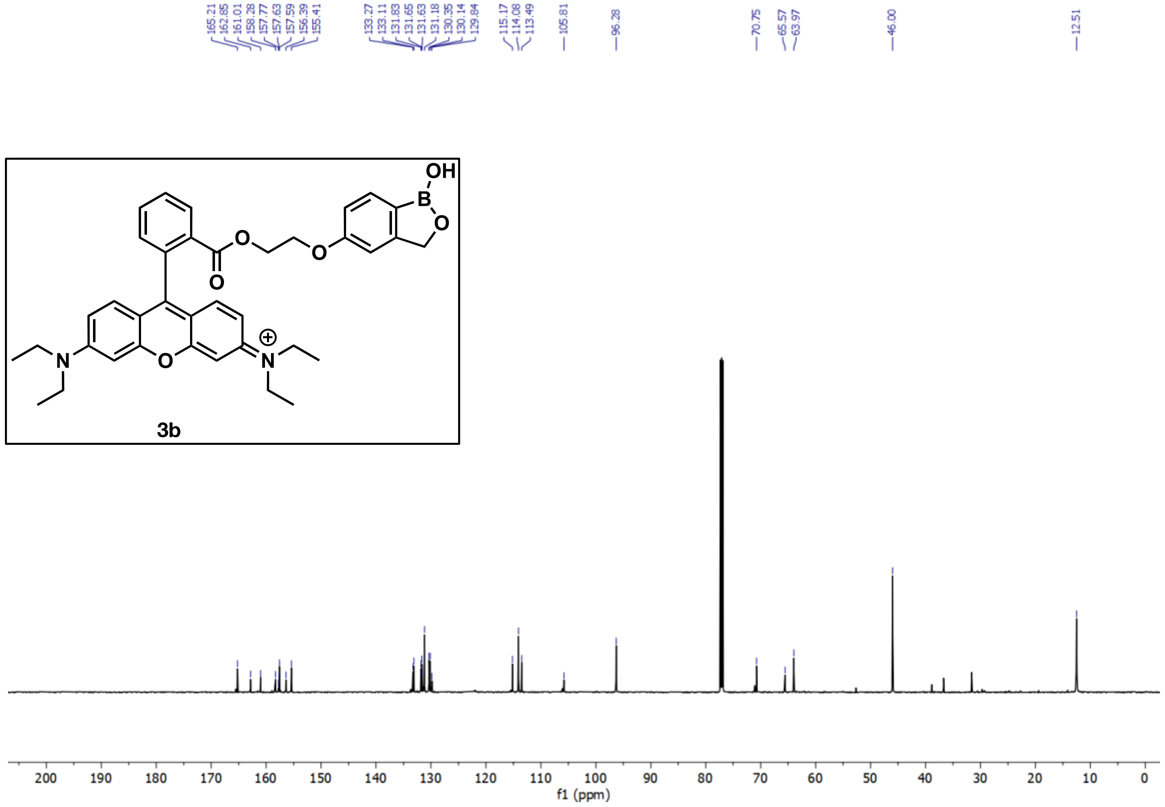
OH
B
O
O
O
O
N
O
N
3b
f1 (ppm)
